# Factors associated with short birth interval in low- and middle-income countries: a systematic review

**DOI:** 10.1186/s12884-020-2852-z

**Published:** 2020-03-12

**Authors:** Juan Pimentel, Umaira Ansari, Khalid Omer, Yagana Gidado, Muhd Chadi Baba, Neil Andersson, Anne Cockcroft

**Affiliations:** 1 CIET/PRAM, Department of Family Medicine, McGill University, 5858 Chemin de la Côte-des-Neiges 3rd Floor, Suite 300, Montreal, Quebec H3S 1Z1 Canada; 2 Facultad de Medicina, Universidad de La Sabana, Campus Universitario puente del común, Chía, Colombia CP 250001; 3 Escuela de Medicina y Ciencias de la Salud, Universidad del Rosario, Carrera 24 # 63 C 69, Bogotá, Colombia; 4 Centro de Investigación de Enfermedades Tropicales (CIET), Universidad Autónoma de Guerrero, Calle Pino s/n Colonia El Roble, 39640 Acapulco, Guerrero Mexico; 5 Federation of Muslim Women Association of Nigeria (FOMWAN), Bauchi, Nigeria

**Keywords:** Birth intervals, Developing countries, Breastfeeding, Pregnancy outcome, Systematic review

## Abstract

**Background:**

There is ample evidence of associations between short birth interval and adverse maternal and child health outcomes, including infant and maternal mortality. Short birth interval is more common among women in low- and middle-income countries. Identifying actionable aspects of short birth interval is necessary to address the problem. To our knowledge, this is the first systematic review to systematize evidence on risk factors for short birth interval in low- and middle-income countries.

**Methods:**

A systematic mixed studies review searched PubMed, Embase, LILACS, and Popline databases for empirical studies on the topic. We included documents in English, Spanish, French, Italian, and Portuguese, without date restriction. Two independent reviewers screened the articles and extracted the data. We used the Mixed Methods Appraisal Tool to conduct a quality appraisal of the included studies. To accommodate variable definition of factors and outcomes, we present only a narrative synthesis of the findings.

**Results:**

Forty-three of an initial 2802 documents met inclusion criteria, 30 of them observational studies and 14 published after 2010. Twenty-one studies came from Africa, 18 from Asia, and four from Latin America. Thirty-two reported quantitative studies (16 studies reported odds ratio or relative risk, 16 studies reported hazard ratio), 10 qualitative studies, and one a mixed-methods study. Studies most commonly explored education and age of the mother, previous pregnancy outcome, breastfeeding, contraception, socioeconomic level, parity, and sex of the preceding child. For most factors, studies reported both positive and negative associations with short birth interval. Shorter breastfeeding and female sex of the previous child were the only factors consistently associated with short birth interval. The quantitative and qualitative studies reported largely non-overlapping results.

**Conclusions:**

Promotion of breastfeeding could help to reduce short birth interval and has many other benefits. Addressing the preference for a male child is complex and a longer-term challenge. Future quantitative research could examine associations between birth interval and factors reported in qualitative studies, use longitudinal and experimental designs, ensure consistency in outcome and exposure definitions, and include Latin American countries.

**Trial registration:**

Prospectively registered on PROSPERO (International Prospective Register for Systematic Reviews) under registration number CRD42018117654.

## Background

Adequately spaced births allow women to recover from previous pregnancies, but both too short or too long intervals lead to adverse maternal, perinatal, neonatal, and child health outcomes [1]. The World Health Organization (WHO) currently recommends an interval between the last live birth and the next pregnancy of at least 24 months [2], a birth interval of 33 months.

A meta-analysis published in 2006 reported that short (< 18 months) and long (> 59 months) intervals between two consecutive pregnancies were associated with preterm birth, low birth weight, and being small for gestational age [1]. Another meta-analysis found an association between a birth interval of less than 24 months and infant mortality [3], and others have reported an association between short birth intervals and schizophrenia in the offspring [4]. Shorter interpregnancy intervals are associated with premature membrane rupture, abruptio placentae and placenta previa, and uterine rupture among women with previous caesarean section [5]. Similarly, very long birth intervals (> 5 years) are associated with adverse maternal outcomes such as pre-eclampsia [6].

A systematic review published in 2012 suggested potential mechanisms for the adverse consequences of short birth interval [7]. These include poor maternal nutritional status and folate depletion, suboptimal lactation for the newborn, cervical insufficiency, infections, sibling competition, incomplete healing of the uterus, and abnormal remodelling of endometrial blood vessels. Short birth intervals may also limit the opportunities for economic development of women and their families [8].

One estimate suggests that around 2 million of the 11 million deaths per year of children under 5 years old could be prevented by avoiding birth intervals of less than 2 years [9]. International bodies such as the WHO and USAID have called for further research and actions to address short birth interval [2, 10].

Short birth interval is more common among women in low- and middle-income countries, where an estimated 17% of married women of reproductive age are reported to have unmet needs for family planning [11]. For example, the 2018 Nigerian Demographic and Health Survey (DHS) reported that 19% of married women have unmet family planning needs [12]. Similarly, the 2013 Nigerian DHS found that 23% of women had a birth interval of less than 24 months for their last two births and 62% had an interval of less than 36 months [13].

Several systematic reviews have examined the consequences of birth interval for maternal and child health outcomes [1, 3, 5]. Although a number of individual studies reported on potentially causal factors associated with short birth interval [8, 14, 15], we are not aware of any systematic review that has examined the determinants of short birth interval. Understanding the actionable factors related to short birth interval is crucial to inform efforts to address the problem, particularly in low- and middle-income countries. We therefore conducted a systematic review to examine the factors associated with short birth interval in low- and middle-income countries.

## Methods

We registered the protocol prospectively on PROSPERO (International Prospective Register for Systematic Reviews) under registration number CRD42018117654 [16]. Our research question was: what are the factors associated with birth interval in low- and middle-income countries? We report our systematic review following the Preferred Reporting Items for Systematic Reviews and Meta-Analyses (PRISMA) guidelines [17]. Our PRISMA checklist is available in Additional file 1.

### Search strategy

Using key terms specific to each database, Boolean operators, and truncators, we developed the search strategy with the collaboration of an experienced librarian from McGill University. We included PubMed, Embase, LILACS, and Popline, which is a database specialized in maternal and reproductive health containing grey literature [18]. Our search strategy is available in Additional file 2.

### Eligibility criteria

We used the following inclusion criteria: (i) experimental or observational studies reporting a measure of association (such as relative risk, odds ratio, or hazard ratio), qualitative studies, and mixed methods studies; (ii) studies in English, Spanish, French, Italian, and Portuguese; (iii) studies conducted in low- or middle-income countries according to the World Bank classification [19]; (iv) exposure: including but not limited to community-based factors, demographic factors, epidemiologic factors, socioeconomic factors, environmental factors, and sociocultural factors; (v) outcome: length of intervals between births to women in the population in any time unit. We did not have restrictions regarding the year of publication.

We excluded theoretical publications, quantitative studies reporting only proportions or *p*-values (without a measure of magnitude of association), studies from high-income countries, studies using “birth spacing” as a synonym for contraception use, and studies exploring factors associated with the interval between marriage and first birth.

### Data collection

Using the open-source systematic review web application Rayyan [20], two independent researchers (JP and UA) screened the titles and abstracts and included only the potentially relevant articles. They resolved discrepancies by discussion and consensus, involving a third party (AC) in case of no resolution. Subsequently, we retrieved the full-text articles of all the selected references and removed the duplicates using EndNote X8.2. JP and UA performed the final selection of studies using an eligibility format based on the elements listed in the search strategy. We piloted this format on 5% of the retrieved studies.

The research team collectively designed the data extraction form based on the variables that would answer the research question, in an iterative process with regular meetings to discuss and update the form. JP and UA independently piloted the data extraction form on 5% of the studies to determine its appropriateness. They then extracted data from the included studies and conducted a cross-check review to verify the quality and accuracy of the extracted data.

We extracted the following data when available: basic study information (title, year of publication, country, authors, type of document, and journal); population and setting (sample size, age range, ethnicity, study setting); study methods (aim, study design, unit of analysis, statistical methods, qualitative methods used, ethical approval); and results (exposure and outcome definition, crude and adjusted measure of association, confidence interval, number of participants with/without the outcome in the exposed/non-exposed groups, qualitative findings).

We planned to conduct a quantitative synthesis only if the included studies were sufficiently homogeneous and of adequate quality. As definition of factors was heterogeneous and study quality was variable, we performed a narrative synthesis of the quantitative findings and an inductive thematic analysis [21] on the results of the qualitative studies.

### Methodological quality

To assess the quality of the included studies, we used the 2018 Mixed Methods Appraisal Tool (MMAT), a quality appraisal instrument for systematic reviews including qualitative, quantitative, and mixed methods studies [22]. Researchers have reported on this tool’s efficiency, reliability [23], and content validity [24]. JP and UA independently performed the quality assessment of each publication.

## Results

**Fig. 1:**
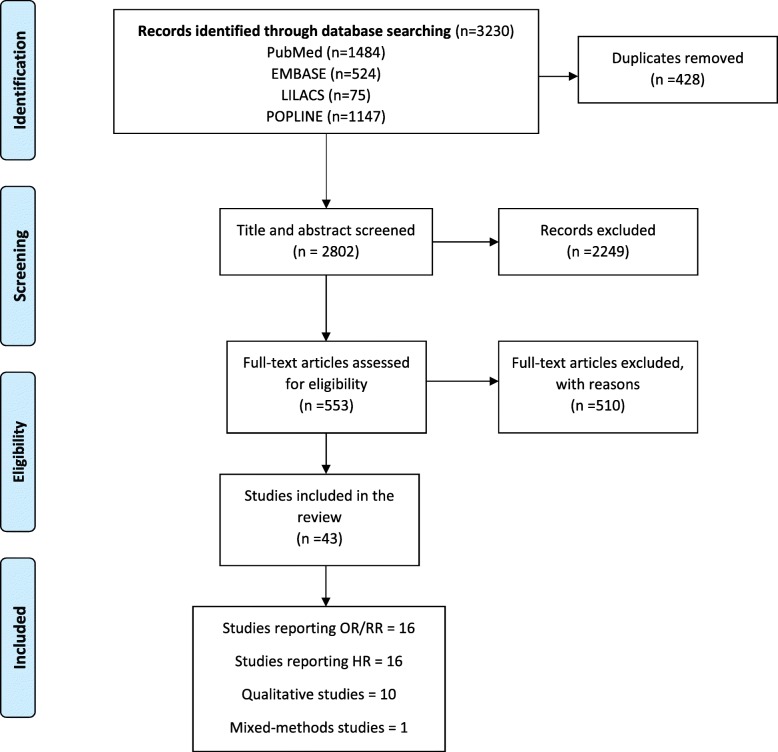
PRISMA flow diagram

From an initial 2802 documents identified by our search, 43 studies remained after screening and assessment (Additional file 3). We categorized these into four subgroups: studies reporting odds ratio (OR) or relative risk (RR); studies reporting hazard ratio (HR); qualitative studies; and mixed methods studies (Fig. 1 and Additional file 4).

**Fig. 2:**
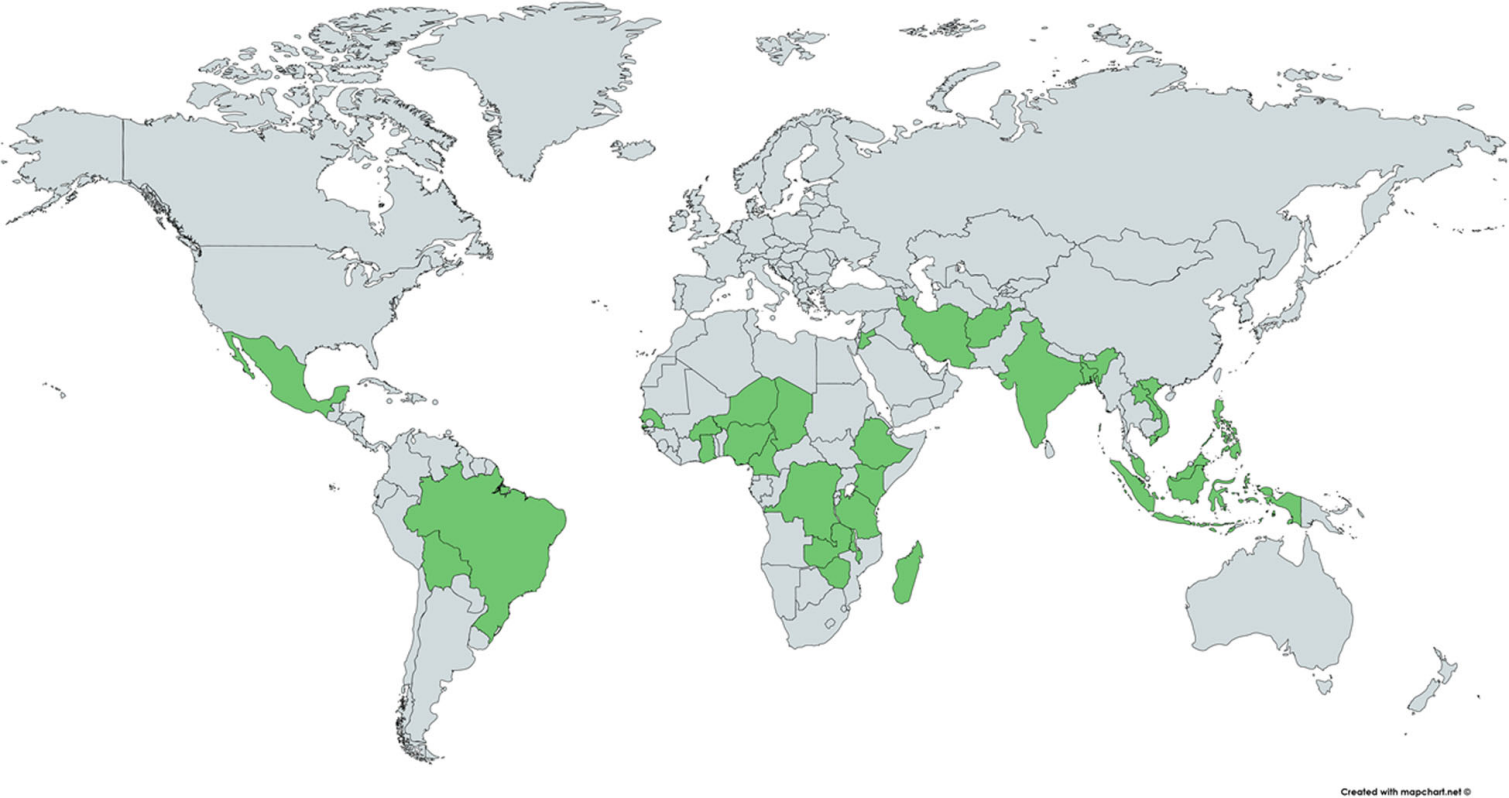
Countries where the studies were conducted. Created and reproduced with permission from the open-source web application mapchart.net [25].

Figure 2 shows the countries where the studies took place. Africa produced the most studies (21/43), followed by Asia (18), and the Americas (four). The countries with the most studies were Nigeria (five studies), Tanzania (five studies), and India (four studies). Some 14 studies were published after 2010, 14 studies between 2001 and 2010, eight studies between 1990 and 2000, and seven studies before 1990. Four documents were reports (two from grey literature) and the remainder were journal articles. Most of the quantitative studies used a cross-sectional design (24/32) and sample sizes ranged widely from 134 to 64,943 (Table 1).

**Table 1:** Characteristics of included studies

First author and year of publication	Country	Sample size	Unit of analysis	Age range/mean (years)	Aim of the study
**Studies reporting OR/RR**					
Abdullah 2018^a^	Bangladesh	4504	Women	15 to 35+	“examines the feasibility of integrating a post-partum family planning intervention package within a community-based maternal and newborn health intervention package, and evaluates the impact of integration on reduction of rates of short birth intervals and preterm births.”
Hailu 2016^b^	Ethiopia	636	Women	Mean 31 (SD ± 5.16)	“assess determinants of interbirth interval among child-bearing age women who have at least two consecutive live births in Arba Minch ZuriaWoreda, SNNP, Ethiopia, 2014.”
Chirwa 2014^c^	Democratic Republic of Congo	7172	Women	15 to 49	“investigate the proportion of short birth intervals at the provincial level among young women in the DRC.”
de Jonge 2014^c^	Bangladesh	5571	Births	NR	“identify predictors of short birth interval and determine consequences of short intervals on pregnancy outcomes.”
Begna 2013^b^	Ethiopia	636	Women	20 to 49	“assess the determinants of inter birth interval among women’s of childbearing age in Yaballo Woreda, Borena zone, Oromia Regional State, Ethiopia.”
Dim 2013^c^	Nigeria	420	Women	20 to 44	“determined the duration of inter-birth interval and the determinants of short inter-birth interval in Enugu, Nigeria.”
Muganyizi 2013^c^	Tanzania	427	Women	15 to 45mean 29.2(SD ± 5.1)	“explored if the use of modern family planning promotes healthy timing and spacing of pregnancy among women seeking antenatal services.”
Exavery 2012^d^	Tanzania	8980	Women	16 to 49	“(1) describe the median level of inter-birth interval (in months), (2) estimate proportions of inter-birth intervals below the recommended minimum inter-birth interval by characteristics of mother and child, and (3) identify factors associated with non-adherence to the recommended minimum inter-birth interval among multiparous women of childbearing age in Rufiji district of Tanzania.”
Fayehun 2011^c^	Nigeria	22,752	Births	15 to 49	“examine the effects of demographic, socioeconomic and socio-cultural factors on birth spacing among Nigerian ethnic groups.”
Ismail 2008^c^	Malaysia	355	Women	Mean 33.5 (SD ± 5.0)	“determine the prevalence and associated factors for short birth spacing among Malay women who delivered at Hospital Universiti Sains Malaysia, Kota Bharu, Kelantan.”
Todd 2008^c^	Afghanistan	4452	Women	Mean 25 (SD ± 5.7)	“assess prevalence and correlates of prior contraceptive use among hospitalized obstetric patients in Kabul, Afghanistan.”
Ngianga-Bakwin 2005^c^	Nine countries in Africa^e^	50,596	Birth intervals	NR	“investigate associations between use of depot-medroxyprogesterone acetate and other reversible contraception and short birth intervals in sub-Saharan Africa.”
Sirivong 2003^c^	Laos	298	Women	15 to 49	“find out whether or not the training of traditional birth attendants had an impact on reproductive health.”
Atkin 1992^d^	Mexico	137	Women (adolescents)	< 18	“identifies and explores selected background, pregnancy, and postpartum predictors of short-interval repeat pregnancy among urban Mexican adolescents who were single when they conceived their first pregnancy.”
Achadi 1991^c^	Indonesia	6826	Birth intervals	NR	“examine the relative impact of breastfeeding and family planning use on birth spacing patterns in two major regions of Indonesia.”
Franca-Junior 1985^c^	Brazil	345	Children	NR	“investigate the interpregnancy interval and its relationship with breastfeeding”(translated from Portuguese).
**Studies reporting HR**					
Blackwell 2015^d^	Bolivia	986	Women	NR	“investigated associations between intestinal helminths and fertility in women.”
Mattison 2015^c^	Tanzania	315	Children	2 to 7mean 4.5 (SD ± 1.6)	“ask whether breastfeeding for more than 2 years is associated with discernible health and well-being benefits to children.”
Erfani 2014^c^	Iran	9071	Women	15 to 49	“study the determinants of change in the timing of births.”
Fallahzadeh 2013^c^	Iran	400	Women	15 to 49	“identify the duration and determinants of inter birth intervals among women of reproductive age in the city of Yazd.”
Singh 2012^c^	India	7624	Women	NR	“see the effect of breastfeeding as a time-varying and time-dependent factor on birth spacing in order to provide input to policy planners.”
Dommaraju 2008^c^	India	64,943	Women	15 to 49	“investigates the complex relationship between marriage age and marital fertility by examining the initiation of childbearing and the transition to higher order births by marriage cohorts in India.”
Hossain 2007^d^	Bangladesh	31,324	Birth intervals	Mean 21.8	“examine the relationship between child mortality and subsequent fertility.”
Ly 2006b^a^	Senegal	134	Children and their moms	3.5 months old babies	“assess the effects of early, short-term food supplementation of infants (from 4 to 7 months of age) on maternal weight change, duration of breastfeeding and birth interval in a rural West African community.”
Gyimah 2005^c^	Ghana	10,975	Women	15 to 49	“[examines] the relative socio-economic vis-a-vis socio-cultural factors on the timing of births.”
Upadhyay 2005^c^	Philippines	1123	Women	26 to 49mean 37	“look at whether women’s status and autonomy affect birth-to-conception intervals.”
Youssef 2005^c^	Jordan	4349	Birth intervals	15–49mean 32.2(SD ± 7.1)	“identify the duration and determinants of interbirth intervals among women of reproductive age in one region of Jordan.”
van Eijk 2004^c^	Kenya	2218	Women	14 to 30+	“studied factors associated with short pregnancy interval (PI) and the effect of PI on birthweight and haemoglobin.”
Hoa 1996^c^	Vietnam	1132	Women	NR	“explore the reproductive pattern of women in rural Vietnam in relation to the existing family planning policies and laws.”
Nair 1996^c^	India	1829	Women	< 35	“examine changes in the timing of birth and the important factors determining birth intervals.”
Adewuyi 1990^c^	Nigeria	8818	Women	NR	“examine regional variations in birth interval length as reported in the Nigerian Fertility Survey and the pattern in the variation of birth interval length at different parities. [...] examination of the correlates of birth interval length in the country.”
Lehrer 1984^c^	Malaysia	1200	Women	< 50	“test the hypothesis that the impact of child mortality on spacing varies across parities.”
**Mixed-methods studies**					
Dehne 2003	Burkina Faso	350	Community members	15–49	“document current trends in knowledge of, attitudes towards, and relating to traditional and modern child-spacing methods in a remote area in northern Burkina Faso.”
**Qualitative studies**					
De Vera 2007	Philippines	7	Couples (husbands and wives)	20 to 47	“describe perceptions of birth spacing among rural Filipino husbands and wives.”
Social & Rural Research Institute 2003	India	34	Focus groups	17 to 30	“understand knowledge, attitudes, behaviors and practices with respect to birth spacing; determine the factors that motivate birth spacing among those who practice spacing; identify barriers to adoption of spacing methods; understanding knowledge, attitudes and practices of health personnel and institutional support towards birth-spacing.”
Dean 1994	Kenya	153 participants	Community groups	NR	“examine the beliefs held and concepts behind childbearing practices in the rural communities of West Pokot District in Kenya and the concrete changes in these practices that have occurred.”
Chad Ministry of Public Health 1992	Chad	16 focus groups with 160 men and women	Focus groups	18 to 40	“1. Learn how Chadian men and women feel about the concept of family wellbeing; 2. Explore men and women’s understanding of modern family planning methods and family well-being, including rumors and misconceptions; 3. Examine the influence of religion on the use of family planning among Chadian men and women; 4. Examine the image Chadian women and men have of a family planning user.”
Kiluvia 1991	Tanzania	50	Focus groups	15 to 35+	“identify persuasive, educational, and appealing family planning messages for radio and print materials. […] To learn why Tanzanian couples choose to space their births.”
Van de Walle 1986	Burkina Faso	80	Women	NR	“revisited 80 women, for a longer description of their postpartum experiences.”
Millard 1984	Mexico	285	Women	> 15	“shows how cultural systems, in addition to biological constraints, shape lactation patterns and endow breastfeeding with social significance.”
Lovel 1983	Zimbawe	204	Women	NR	“women with at least one child under five were asked about reasons for birth spacing in their parents’ generation.”
Adeokun 1982	Nigeria	NR	Families	NR	“marital sexual relationships (MSR) and the timing of the next child among the Ekiti and Ikale sub-groups of the Yoruba.”
Adeokun 1981	Nigeria	24	Families	NR	“investigate the patterns of maternal and child care, the parents’ perception of the timing of various milestones in the development of their children, and to seek the links between marital sexuality, child development and the timing of a next child (other than the first).”

*NR* not reported

^a^Experimental

^b^Case-control

^c^Cross-sectional

^d^Cohort/longitudinal

^e^Burkina Faso, Cameroon, Ghana, Kenya, Madagascar, Malawi, Niger, Tanzania, Zambia

### Studies reporting OR/RR

Some 11 studies reported factors associated with short birth interval after adjusting for confounders. The most commonly reported factors were age of the mother (seven studies), education of the mother (six), and contraception use (five). Five studies defined short birth interval as < 24 months, while two studies used < 33 months and < 36 months, respectively (two studies did not provide their definition of short birth interval). For all factors but length of breastfeeding (three studies) and sex of the previous child (two studies), researchers reported mixed results. Table 2 shows the associations reported between factors and short birth interval.

**Table 2:** Adjusted OR/RR and confidence interval of factors significantly associated with birth spacing

Category / subcategory	Author / year of publication	Factor	Outcome (length of birth interval in months)	Adjusted effect size	Confidence interval 95%
Age					
At first pregnancy (years)	de Jonge 2014	18.3 vs 18.6	< 33	**0.95**	**0.92–0.98**
At delivery of the last child (years)	Hailu 2016	17–24 vs > 34	< 36	0.98	0.36–2.66
		25–29 vs > 34		0.9	0.40–2.0
		30–34 vs > 34		**2.58**	**1.08–5.15**
	de Jonge 2014	22 vs 22.88	< 33	**1.11**	**1.08–1.15**
Of the mother (years)^a^	Chirwa 2014	15–19 vs 45–49	< 25	**2.51**	**1.56–4.04**
		20–24 vs 45–49		**1.79**	**1.27–2.52**
		25–29 vs 45–49		1.3	0.93–1.84
		30–34 vs 45–49		1.25	0.89–1.76
		35–39 vs 45–49		1.05	0.74–1.5
		40–44 vs 45–49		0.99	0.69–1.44
Of the mother (years)^b^	Begna 2013	20–24 vs 25–29	< 36	1.36	0.53–3.48
		30–34 vs 25–29		0.68	0.39–1.17
		35–39 vs 25–29		**0.31**	**0.17–0.6**
		40–44 vs 25–29		**0.22**	**0.10–0.49**
		45–49 vs 25–29		0.39	0.15–1.01
	Muganyizi 2013	30 vs 15–29	< 24 or > 60	1	0.5–1.7
	Exavery 2012	15–19 vs 45–49	< 33	**13.65**	**9.63–19.35**
		20–24 vs 45–49		**4.3**	**3.16–5.86**
		25–29 vs 45–49		**2.4**	**1.77–3.26**
		30–34 vs 45–49		**2.07**	**1.52–2.8**
		35–39 vs 45–49		**1.64**	**1.21–2.24**
		40–44 vs 45–49		1.31	0.95–1.83
	Ismail 2008	One-year increase in age	< 24	**0.86**	**0.8–0.92**
	Ngianga-Bakwin 2005	< 21 vs > 21 and < 35	< 24	**0.58**	**0.55–0.62**
		> 35 vs > 21 and < 35		**0.67**	**0.62–0.72**
Of the woman’s own mother at first pregnancy (years)	Atkin 1992	11–17 vs > 17	< 24	5.1	Not provided
Breastfeeding					
Duration (months)	Hailu 2016	12–23 vs > 23	< 36	**60.19**	**31.61–114.59**
	Begna 2013	< 25 vs > 24	< 36	**30.81**	**6.97–136.19**
	Ismail 2008	< 12 vs > 11	< 24	**6.18**	**3.59–10.62**
Type	Chirwa 2014	Exclusive breastfeeding vs Mixed	< 25	1.08	1–1.17
		Never breastfeeding vs Mixed		1.07	0.99–1.15
	Ngianga-Bakwin 2005	Exclusive breastfeeding vs No breastfeeding	< 24	**0.67**	**0.58–0.78**
		Mixed feeding vs No breastfeeding		**0.86**	**0.82–0.90**
Contraception					
Method	Chirwa 2014	Not using contraception vs Modern method	< 25	0.97	0.85–1.08
	Ngianga-Bakwin 2005	Using injections vs Using other methods	< 24	**1.23**	**1.1–1.38**
	Atkin 1992	Postpartum IUD: No/Yes	< 24	26.34	Not provided
Use	Hailu 2016	No vs Yes	< 36	**3.01**	**1.68–5.39**
	Muganyizi 2013	No vs Yes	< 24 or > 60	**1**	**0.6–1.8**
	Begna 2013	No vs Yes	< 36	**5.91**	**4.02–8.69**
	Ismail 2008	No vs Yes	< 24	**3.95**	**2.21–7.05**
	Ngianga-Bakwin 2005	No vs Yes	< 24	**0.88**	**0.82–0.93**
	Todd 2008	Prior contraceptive use: Yes/No	Longer mean birth interval (2.21 ± 0.79 Vs 2.01 ± 0.87 years)	**1.25**	**1.12–1.40**
Education of the mother					
	Hailu 2016	No formal education vs Has formal education	< 36	**3.4**	**1.8–6.43**
	Chirwa 2014	No education vs Secondary or higher	< 25	1.08	0.97–1.19
		Primary education vs Secondary and higher		1.06	1–1.16
	de Jonge 2014	Secondary or above vs None or primary education	< 33	**1.26**	**1.09–1.45**
	Begna 2013	No formal education vs Formal education	< 36	**1.89**	**1.15–3.37**
	Muganyizi 2013	Primary vs No education	< 24 or > 60	1	0.2–4.6
		Secondary or above vs No education		1.6	0.3–7.3
	Exavery 2012	Never been to school vs Secondary/higher	< 33	**1.27**	**1.01–1.60**
		Primary vs Secondary/higher		1.09	0.87–1.37
	Ngianga-Bakwin 2005	No education vs Secondary education or higher	< 24	**1.16**	**1.06–1.26**
		Primary education vs Secondary education or higher		**1.11**	**1.03–1.20**
	Sirivong 2003	Literate vs Illiterate	Birth spacing (length not specified)	**0.27**	**0.08–0.84**
Marital status					
	Muganyizi 2013	Not in marriage vs In marriage	< 24 or > 60	0.9	0.4–2.1
	Exavery 2012	Previously married (widowed or divorced) vs Married	< 33	**0.56**	**0.48–0.66**
		Single vs Married		**0.64**	**0.57–0.73**
	Atkin 1992	Women who were in a legal or consensual union at 5 months: Yes / No	< 24	6.9	Not provided
Miscellaneous					
Intervention	Abdullah 2018	**Intervention**: integrated post-partum family planning and maternal and newborn health. **Control**: maternal and newborn health only.	< 24	**0.81**	**0.69–0.95**
Occupational group	de Jonge 2014	Tea garden resident: Yes vs No	< 33	**1.41**	**1.07–1.87**
Religion		Other vs Muslim		**0.68**	**0.53–0.87**
Time period	Ngianga-Bakwin 2005	1998–2001 vs 1991–1993	< 24	**0.9**	**0.84–0.95**
Occupation					
Husband	Begna 2013	Daily worker vs Animal husbandry	< 36	**2.19**	**1.01–4.79**
		Farmers vs Animal husbandry		0.49	0.24–1
		Merchant vs Animal husbandry		0.72	0.36–1.43
		Others vs Animal husbandry		1.17	0.47–5.92
Mother	Hailu 2016	Farmer vs Others	< 36	2.68	0.31–23.23
		Housewife vs Others		1	0.17–5.86
		Merchant vs Others		1.46	0.16–13.24
	Muganyizi 2013	Employment Business vs Salary employment	< 24 or > 60	1.1	0.6–2.1
		Employment Housewife/others vs Salary employment		1.6	0.7–3.4
Parity (children)					
	de Jonge 2014	> 3 vs 1	< 33	**0.28**	**0.19–0.41**
		2 vs 1		**0.53**	**0.44–0.63**
		3 vs 1		**0.38**	**0.29–0.51**
	Muganyizi 2013	> 3 vs 2	< 24 or > 60	1.8	0.9–3.7
		3 vs 2		0.9	0.5–1.5
	Begna 2013	2 vs > 4	< 36	**3.73**	**1.50–9.25**
		3&4 vs > 4		**2.69**	**1.23–5.92**
	Exavery 2012	> 3 vs 2	< 33	**2.54**	**2.25–2.85**
		3 vs 2		**1.29**	**1.19–1.40**
	Ismail 2008	Parity (no further explanation)	< 24	**1.46**	**1.22–1.76**
Previous pregnancy					
Outcome	de Jonge 2014	Adverse outcome of any previous pregnancy: Yes / No	< 33	**2.1**	**1.83–2.40**
	Muganyizi 2013	Immediate past pregnancy loss: Yes / No	< 24 or > 60	**2.5**	**1.3–4.7**
	Exavery 2012	Birth: Multiple vs Singleton	< 33	**0.74**	**0.57–0.96**
	Ismail 2008	History of abortion: Yes vs No	< 24	**0.09**	**0.02–0.34**
Place of delivery	Hailu 2016	Health institution vs Home	< 36	1.53	0.61–3.8
	Exavery 2012	Elsewhere vs Health facility	< 33	**1.85**	**1.71–2**
Planned	Hailu 2016	No vs Yes	< 36	1.44	0.9–2.61
Sex of the previous child					
	Hailu 2016	Female vs Male	< 36	**6.79**	**3.65–12.63**
	Begna 2013	Female vs Male	< 36	**1.72**	**1.17–2.52**
Socioeconomic level					
Household assets^c^	de Jonge 2014	0–3 vs > 3	< 33	**1.42**	**1.22–1.65**
Income	Hailu 2016	Wealth index: Fourth vs Richest	< 36	**3.96**	**1.41–11.13**
		Wealth index: Middle vs Richest		**3.98**	**1.39–11.38**
		Wealth index: Second vs Richest		**6.46**	**2.26–8.48**
		Wealth index: Poorest vs Richest		**14.33**	**4.65–44.15**
	Chirwa 2014	Low vs High	< 25	0.98	0.8–1.01
		Middle vs High		**0.86**	**0.77–0.94**
	Ngianga-Bakwin 2005	Low vs High	< 24	1.18	**1.10–1.26**
		Middle vs High		**1.25**	**1.17–1.34**
Place of residence	Chirwa 2014	Rural vs Urban	< 25	1.07	0.97–1.13
	Exavery 2012	Rural vs Urban	< 33	1.04	0.95–1.13
	Ngianga-Bakwin 2005	Urban vs Rural	< 24	**0.85**	**0.79–0.9**

Significant results are shown in bold

^a^When the study was conducted

^b^Did not specify a definition of age of the mother

^c^Electricity, radio/tape recorder, fan, TV, fridge, phone, generator and bicycle

Seven studies found an association between a short birth interval and a younger age of the mother. Among these publications, two studies explored age at last delivery, one study explored the age of the mother at the moment of the study, and the remaining studies did not specify a definition of age of the mother. Two studies reported an association between a short birth interval and older age of the mother (one study explored age at first pregnancy).

Three studies found an association between short birth interval with shorter duration of breastfeeding. One study found more short birth interval with no breastfeeding as opposed to exclusive or mixed breastfeeding.

Three studies reported an association between a short birth interval and no contraception use, while one publication reported the contrary. The latter study used data from Demographic and Health Surveys from nine sub-Saharan African countries between 1991 and 2001.

Five studies reported more short birth interval with less education, but one study reported more short birth interval with more education. Two studies reported an association between short birth interval and less parity, while one study reported the contrary.

Two studies reported more short birth interval with lower income, while one study reported the contrary. Similarly, a study reported an association between short birth interval and fewer assets. One study reported more short birth interval for people living in rural areas compared with people living in urban settings.

Three studies reported an association between short birth interval and an adverse outcome of a previous pregnancy, but one study found longer birth interval after a previous abortion. One study found more short birth interval after a singleton birth compared with after a multiple birth. One study found more short birth interval when the place of delivery of the index child was not a health facility. Two studies found more short birth interval when the sex of the previous child was female.

An experimental study found more short birth interval with maternal and newborn health care only, compared with integrated post-partum family planning and maternal and newborn health care. Four studies reported other factors associated with birth intervals such as marital status of the mother, occupation of husband, place of residence, year, and religion (Table 2).

After adjusting for confounders, six studies reported non-significant associations between different factors and short birth interval. The studies explored age of the mother at the moment of the study (four publications), education of the mother (three), contraception method, marital status of the mother, occupation of the mother, and place of residence (two publications each category). Other factors examined included contraception use, age at last delivery, age of the woman’s mother at first pregnancy, type of breastfeeding, occupation of the husband, parity of the mother, delivery place of the previous pregnancy, planned previous pregnancy, and income level (one publication each category).

### Studies reporting HR

Interpretation of time-to-event data is challenging since the nature of association may change over time. For example, more education can lead to longer birth intervals in the first two births, but it may have the opposite effect for subsequent births. We have summarized the authors’ overall conclusions for each study (Additional file 4).

Among the 16 studies reporting HR, researchers most frequently reported on factors such as education (eight studies), previous pregnancy outcome (seven), age of the mother (six), breastfeeding (four), and socioeconomic level (three). All the studies reported mixed results for all factors except for breastfeeding, for which four studies found more short birth interval with shorter breastfeeding in all the pregnancies.

Three studies reported shorter birth intervals with a younger age of the mother, while one study reported the contrary. Two studies that looked at age at marriage and birth interval reported mixed results, and one study found shorter birth intervals with younger age at first birth.

Four studies found shorter birth intervals with lower education of the mother, while three studies found the contrary, and two other studies found mixed results. Five studies found shorter birth intervals with adverse outcomes in any previous pregnancy, while one study found the opposite. Two studies found mixed results.

One study reported a shorter birth interval with roundworm (Ascaris lumbricoides) infection but extended interbirth intervals with hookworm infection. Another study found longer birth intervals among women with HIV infection.

One study reported shorter birth intervals among married couples, while another study found shorter birth intervals with shorter duration of the marriage. Regarding socioeconomic level, a study found shorter birth intervals with lower income, another study found a shorter birth interval with rural residence, and a third study found mixed results (place of residence).

Six studies found shorter birth intervals with less parity, postpartum amenorrhea less than 6 months, when the previous child is a girl, not using modern contraception, when religion is Catholic, farmer as a profession, and less female decision-making autonomy. An intervention study found longer birth intervals with early short-term infant supplementation. A study found mixed results on birth interval for media exposure and employment status of the women.

### Qualitative and mixed-methods studies

We identified nine themes among the findings of the qualitative studies. The leading theme was local concepts and practices (14 codes), followed by modernization (eight), communication (six), religion (four), breastfeeding, health concerns, knowledge and attitudes, miscellaneous (two codes each theme), and autonomy (one).

According to the participants of the studies, the observance of local traditions prevents short birth interval. Examples include *agbon*, described as a “female body odor after parturition” that is to be respected before restarting sexual activity; *apa*, which requires avoiding a conflictive situation between children if spaced too closely; and *ratat*, a traditional period of abstinence. Researchers also reported the influence of local medicine people (hilots, marabu), traditional medicine, use of amulets, talismans, and cords, coitus interruptus, polygamy, and social taboo on the length of birth interval.

Modernization was another concept reported by researchers. The participants of the studies explained that modernization introduces social changes influencing birth intervals such as loss of culture and traditions, men staying at home more often, availability of health services, education, and food, and changes in religion and beliefs. Availability of health services and infrastructure, and family planning education prolong birth interval, while the rest of the factors related to modernization promote short birth interval.

According to the participants of the qualitative studies, birth interval was also affected by communication between couples and families, the influence of local media and the society, and observation of other parents. Concerns for maternal and child health were mentioned as tending to increase birth interval, as was knowledge about contraception methods.

Some participants mentioned that breastfeeding prolongs birth interval, while other participants questioned its effectiveness as a means of preventing an early next pregnancy. Finally, Catholicism and Hinduism were considered to shorten birth spacing, and women’s autonomy, drought, and war were also mentioned to affect the timing of births. Table 3 gives a list of themes, codes, and quotations.

**Table 3:** Factors associated with birth spacing from qualitative studies

Author and year of publication	Theme	Factor	Quotation	Page
Social & Rural Research Institute 2006	**Autonomy**	Lack of decision-making power	“Lack of decision-making powers among the women due to the patriarchal structure of the family that gives the man the reins of power.”	4
Millard 1984	**Breastfeeding**	Breastfeeding does not work	“When the issue of lactation contraception is raised with village women, they deny its effectiveness, confidently and laughingly pointing to their own repeated experiences of returning to pregnancy while breastfeeding. They volunteered information no about the return of menses in relation to the probability of conception. Of the women queried about lactation contraception, only one woman in Amanalco stated that she thought breastfeeding probably did reduce the chance of a rapidly ensuing pregnancy.”	9
Adeokun 1982		Breastfeeding works	“I do not worry my mind about pregnancy, we keep on having sex and as long as she keeps on breast-feeding nothing can happen. Once she has stopped and her period has returned, then we know it is time for her to become pregnant.”	10
De Vera 2007	**Communication**	Couple	“The subcategory, lack of communication, was identified as one of the reasons that couples did not space births.”	242
Adeokun 1982		Family	“The openness of Yoruba families encourages kin and in-laws to influence a couple’s decisions about child rearing and the timing of the next child.”	12
De Vera 2007			“Family and friends influenced couples’ decisions about birth spacing. Some participants received advice on birth spacing from relatives and friends.”	244
Social & Rural Research Institute 2004			“Familial and cultural factors that determine the extent of support lent by the family (or the lack of it) to the woman. Given the nature of the Indian society, the influence of the peers and the community is critical.”	4
De Vera 2007		Media	“The popular media (print and broadcast media) may exert influence on couples’ decisions about spacing births in some communities.”	244
		Observation	“Whereas others observed that their relatives went through hard lives because of too closely spaced pregnancies.”	244
Social & Rural Research Institute 2009		Social	“Fear of social disapproval [is a barrier to adoption of spacing].”	4
Kiluvia 1991		Women’s responsibility	“Communication between spouses’ partners on child spacing was not necessary. Reasons varied with age and gender.[...]. Married men also frequently saw no reason to talk to their wives, because they felt the number and timing of births was solely their responsibility.”	7
Chad Ministry of Public Health 1992			“While Chadians of both sexes agreed that ideally both husband and wife should decide to use child spacing together, there was consensus that it was ultimately a woman’s responsibility to ensure that children were spaced.”	8
Van de Walle 1986	**Health concerns**	Child health concerns	“If you have a child in your arms and you become pregnant again, it is not good. The child is tired and you, who are with a belly, are tired also... the child in your womb suffers too. Everyone will suffer, because you must feed the other one in addition to this one. The one in your womb will be tired too. You know that if there is no one to take care of the oldest one you will be obliged to carry it on top of your pregnancy.”	17
		Maternal health concerns	“Question. When do you want the next child? Answer: Even if it takes 3 or 4 years, I will be happy. Question: Why do you want to wait? Answer: Because I am old and a lot of blood poured out of my body during my deliveries. Can the same blood flow back into my body so rapidly?”	16
Social & Rural Research Institute 2003	**Knowledge and attitudes**	Attitudes of the women	“Personal factors driven primarily by the attitude of the woman to self and spacing.”	4
		Knowledge	“Ignorance of methods available and negligent attitude towards the concept of spacing per se that is catapulted either by the negative word of mouth or bad personal experiences.”	
Dehne 2003	**Local concepts and practices**	Abstinence norms	“Women adhering to Gurmance traditional religion reported either 24 or 36 months’ taboos, while Muslims reported much shorter norms. Most Hamallists (and the few Wahabiya) women reported a 40-day norm as prescribed in the Koran, while many ‘moderate’ Muslims reported intermediate norms of 2–5 months.”	60
Van de Walle 1986		Afraid of being mocked	“As to the women, they crave for a child after 2 years, they are afraid of being mocked because they are finished with childbearing.”	28
Adeokun 1981		Agbon (female body odor)	“The condition called agbon or female body odor after parturition. Once the period of agbon is over, sexual activity is commenced. The interbirth interval is thus a function of the length of agbon, the length of postpartum amenorrhoea, and the practice of pregnancy prevention after agbon has ended.”	14
Dehne 2003		Amulets, talismans, and cords	“The use of amulets, talismans, and cords […] for instance in cases where women felt unprotected against an early pregnancy or guilty after having resumed sexual relations shortly after a preceding birth.”	60
Adeokun 1981		Apa (conflict between children)	“The other Ikale strategy starts from the notion of apa, that is, the principle that if a surviving child is less than 1 year old and another pregnancy occurs, a conflict situation arises between the survival of the nursing child and the survival of the foetus. In order to avoid the conflict, the marital sexual relationship of the parents may be organized in such a way as to prevent the undesirable pregnancy.”	14
Millard 1984		Coitus interruptus	“Conjugal disruption is more frequent in Tepetlaoxtoc and may be a contributing factor; possibly conscious efforts are more often made to birth extend intervals through coitus interruptus.”	7
De Vera 2007		Hilots (abortion for birth spacing)	“Most participants said that abortion was common and a de facto method for spacing births. Because abortion is illegal in the Philippines, women sought hilots (unlicensed lay abortionists, midwives, and masseuses) to perform an abortion.”	243
Kiluvia 1991		Honoured tradition	“We were strongly advised by our parents to take care of the baby for 2 years, then from here you can start thinking about having another baby. We were strictly warned of having too closely spaced children.”	17
Chad Ministry of Public Health 1992			“Child spacing was seen as a tradition that protected children’s and women’s health.”	ii
Lovel 1983		Local knowledge	“These results show that in a traditional society the health benefits of birth spacing to mother and child are clearly well-known.”	162
Dehne 2003		Marabu	“When this was becoming difficult, because my husband no longer agreed to abstain, I went to see a Marabu who gave me an amulet.”	60
Dean 1994		Polygamy	“The influence of the post-partum abstinence period on birth interval and fertility is important. As outlined, men traditionally visit several wives, living far apart, each for a few months at a time, and this helps to sustain the post-partum abstinence period.”	1581
		Ratat (traditional abstinence)	“Results of this study found that there was a traditional form of contraception throughout the district, called ratat or rotow in most areas.”	
Lovel 1983		Social taboo	“More than a quarter of the families (rural 26%, semiurban 30%) said that in their parents’ generation people were ashamed of having children too close together because it was not accepted in the culture.”	161
Van de Walle 1986			“Ah, two years [between children] is good. Certain women don’t reach 2 years. Certain women whose child is not yet walking become pregnant. I cannot understand that... If you have a child in your arms, and become pregnant, don’t you know that you are humiliated [loose face]?”	16
Kiluvia 1991			“Many of the older women felt that engaging in sexual intercourse while the mother is still breastfeeding is taboo.”	18
Adeokun 1982		Traditional medicine	“The other half depends on the use of herbs and traditional devices in the prevention of pregnancy.”	11
De Vera 2007			“Five of the seven women admitted using some medicinal herbs and roots to stop pregnancy or stimulate menstruation.”	243
Kiluvia 1991			“Young men also knew few details of how traditional methods work, but they had heard about the traditional use of herbs and abstinence for birth spacing. [...] Among the most frequently mentioned methods were abstinence; “pigi” and “fungo,” in which a traditional healer ties twigs from a special tree or a “medicated” piece of cloth around a woman’s waist; herbal potions; and douching.”	9
De Vera 2007	**Modernization**	Family planning education	“The health center teaches the use of the modern methods of contraception such as pills and IUD.”	244
Social & Rural Research Institute 2005		Institutional infrastructure	“Institutional infrastructure provided to the populace has also played a decisive role in adoption of spacing methods.”	4
Dehne 2003		Loss of culture	“Many changes have occurred in local customs. Today, the women do not abstain for as long as we used to do.”	61
Dean 1994			“Other major factors thought to cause a decrease in child spacing were the loss of tradition generally, the loss of ratat and the loss of the post-partum abstinence period.”	1581
		Men stay at home more	“With the general degeneration of the traditional society the “men staying at home more” is likely to result in the decrease of the post-partum abstinence period.”	Fig. 8, page 1582
		More food	“When asked specifically what women thought had caused the decrease in birth interval they gave several reasons over and above those relating to traditions, the most important of which was increase in food availability.”	1578
		More health services	“More health services [cause a decrease in child spacing].”	1579
		More hygiene	“More hygiene [causes a decrease in child spacing].”	Fig. 4, page 1579
Dehne 2003		Religion	“In former times, couples waited for one to two year(s) before resuming sexual contacts. Now the waiting period is 2 months or even 40 days.[...] These changes have all occurred because of the increasing influence of religion. Many people listen to the Marabu now and attend Koranic schools.”	60 & 61
Dean 1994	**Other**	Less drought	“Less drought [causes a decrease in child spacing].”	Fig. 4, page 1579
		Less war	“Less war [causes a decrease in child spacing].”	
De Vera 2007	**Religion**	Catholicism	“However, one mother stated that because of the religious saying, “children are gifts from God,” couples end up having many children because they do not have a choice but to accept them.”	243
Adeokun 1982		God’s will	“Chance or God’s will in the avoidance of an inconvenient pregnancy.”	10
Social & Rural Research Institute 2008		Hinduism	“Religious prohibitions dictated by certain scriptures have led to believers not subscribing to spacing.”	4
Chad Ministry of Public Health 1992		Religion	“Religion was also used as a reason by some men, in both Sarh and N’Djamena, for not practicing birth spacing because children were a gift from God.”	v

### Meta-analysis

We decided not to conduct a meta-analysis. The included quantitative studies were very heterogeneous in their definitions of exposures and outcomes, and most used a cross-sectional design with variable approaches to dealing with potential confounders.

### Quality assessment

The quality of most documents ranked as medium (23 documents), followed by high (16 documents), and low (four documents). For information about the quality assessment please see Additional file 5.

## Discussion

Our systematic review shows two factors consistently associated with short birth interval: shorter breastfeeding and a female previous child. Younger age of the mother, less education of the mother, a negative outcome of the previous pregnancy, and lower socioeconomic status were often associated with short birth interval, although some studies reported the opposite. The quantitative studies examined a limited number of factors that could be easily included in a questionnaire.

### Quantitative findings

A contraceptive effect of breastfeeding has long been recognised to prolong birth interval. Breastfeeding causes lactational amenorrhea because the suckling stimulus downregulates hypothalamic gonadotropin-releasing hormone secretion and the production of luteinizing hormone [26].

In 1988, an international group of scientists met at Bellagio, Italy [27], with the support of the WHO, the Rockefeller Foundation, and Family Health International. The group discussed the role of breastfeeding in family planning and concluded that lactational amenorrhoea can be used as a method of contraception. Institutions have followed this advice for decades. The 2017 UK Faculty of Sexual & Reproductive Healthcare Guideline on Contraception After Pregnancy [28] stated that “women may be advised that, if they are less than 6 months postpartum, amenorrhoeic and fully breastfeeding, the lactational amenorrhoea method (LAM) is a highly effective method of contraception.”p21.

Despite current efforts to promote breastfeeding worldwide, early weaning is still common and few children receive exclusive breastfeeding by the age of 6 months [29]. A recent review conducted by Bellù [30] found that breastfeeding support is complex and involves individual, structural, and environmental factors. A better understanding of the factors associated with short breastfeeding is needed. Our review did not aim to identify factors associated with a shorter duration of breastfeeding; this issue should be addressed in future research.

Preference for a son is widespread in North Africa, East and South Asia, and the Middle East [31]. In these regions, people reportedly prefer sons because they earn more, they perpetuate the family line, and they are recipients of the family inheritance [32]. One of the included studies [8] commented that Ethiopian families see a son as an economic asset.

Studies reported mixed results for associations with a negative outcome of the previous pregnancy, age of the mother, education, and socioeconomic level, although with a preponderance of findings in one direction in each case. Researchers conducting the studies in this review often reported an association between adverse outcome of the previous pregnancy, including abortion, pregnancy loss, and multiple birth, and short birth interval. The author of one of the included studies [15] suggested that an adverse outcome might influence women to hurry into the next pregnancy without fully recovering from the last pregnancy. Early researchers on child mortality and fertility described this phenomenon as replacement: “replacement would be the response to experienced mortality [ …] If children die very young and the mother can have another child, the same life cycle can be approximated by replacement.” [33] p164

Most studies found more short birth interval with a younger age of the mother. Younger women are generally more fertile and more sexually active; very young women are likely to be economically disadvantaged, and may have less access to and use of modern contraception, which can explain this association [34–36], though the way this plays out will depend on the local context of each region. Older women are likely to have reached their desired family size and are less fertile, and therefore prone to prolong birth intervals [37, 38].

Most studies reported that less-educated women have more short birth interval. Hailu [8] explains that educated women have a better-informed decision-making process, have greater autonomy, and use higher quality health care services. One study, however, reported opposite results. Highly educated women tend to delay their first pregnancy [39] and RamaRao et al. [37] hypothesized that educated women may want to compress motherhood into fewer years and therefore are likely to have shorter birth intervals.

Most studies in our review found more short birth interval among economically underprivileged women. Hailu [8] suggests that wealthier women have better access to health care information, services, and supplies and therefore, can apply modern contraception to prolong birth intervals.

Similarly, most studies reported expected associations between longer birth intervals with modern contraception use. One cross-sectional study based on DHS surveys reported opposite results [40] and the authors suggested this association between short birth interval and modern contraceptive use could have been due to temporality bias, such that women who had experienced an unintended short birth interval were then preferentially motivated to use modern contraception.

Some of the studies in this review failed to find significant associations between age of the mother, education, contraception method, marital status, occupation, and place of residence, and short birth interval. Together with the fact that some studies showed associations in the opposite direction to the prevalent findings, this suggests that associations with factors such as age and education of the mother are not universal and may be highly dependent on the particular context.

### Qualitative findings

Qualitative studies explored the perceived effects of local concepts and practices not considered in quantitative studies. For example, in Burkina Faso [41] and Kenya [42], studies reported loss of traditional concepts and practices that prolong birth intervals, as a result of modernization. These qualitative findings could help to explain some associations in quantitative studies. For example, it is possible the associations between higher education and higher socioeconomic level and short birth interval could be partly explained by loss of traditions, as more privileged women may report higher levels of acculturation [43].

Qualitative studies considered other factors that were not examined in quantitative studies. Participants in qualitative studies mentioned that communication between couples and families, the influence of local media and the society, and observation of other parents could influence birth intervals. None of the included quantitative studies attempted to explore associations between these factors and birth interval.

Quantitative studies reported a consistent association between longer breastfeeding and longer birth interval. However, participants in one qualitative study questioned the effectiveness of breastfeeding as a means to prolong birth interval, presumably based on their own experiences [44]. The WHO cautions that breastfeeding as the sole method of contraception does not fully protect against new pregnancies; 5–10% of women with lactation amenorrhea nevertheless become pregnant [45].

### Strengths and limitations

Strengths of our study include a broad search strategy guided by a librarian, inclusion of five languages, no year limit, and inclusion of quantitative, qualitative and mixed-methods research, and grey literature.

We chose not to conduct a meta-analysis due to considerable heterogeneity of study design, study quality, population characteristics, and outcome and exposure definitions. We noted the concerns of Egger and co-authors [46] that (i) residual confounding and selection bias are common in observational studies; (ii) meta-analyses of observational data may yield precise but spurious results; (iii) quantitative synthesis should not be a prominent component of systematic reviews of observational studies. Although commonly performed, meta-analysis of observational studies has been criticized and some authors have suggested that this practice should be abandoned [47].

As with all systematic reviews, our results and conclusions are limited by the quality of the original studies. Almost all the included quantitative studies were observational, with concerns about residual confounding even after multivariate analyses [46], and temporality [48] bias, not knowing if the exposure preceded the outcome.

The variability of the definition of short birth interval (24, 33, and 36 months) limits the conclusions of our study. The current WHO recommendation of an optimal birth interval [2] is based on a large body of observational studies published before 2006, most of them coming from low- and middle-income countries. In other settings such as developed countries, a birth interval between 24 to 33 months may not be considered short [49].

## Conclusions

We found two factors consistently associated with short birth interval: shorter breastfeeding duration and a female previous child. Promotion of breastfeeding could help to reduce short birth interval, and has many other benefits [50]. Addressing the preference for a male child is a complex and longer-term challenge.

The quantitative and qualitative studies yielded different and complementary findings. This highlights the potential value of mixed-methods research. Quantitative researchers should look for ways to investigate factors such as local knowledge and practices, modernization, and communication. Future research should use longitudinal and experimental designs, aim for consistency in outcome and exposure definitions, and include Latin American countries.

## Supplementary information

**Additional file 1.** PRISMA 2009 Checklist. Filled PRISMA checklist showing 27 items and the page reporting each item.

**Additional file 2.** Search strategy. Key terms specific to each database, Boolean operators, and truncators.

**Additional file 3.** Full references of the records included in our study. List showing the full references of the documents included in our study.

**Additional file 4.** Studies reporting HR. Table showing the findings of studies reporting HR.

**Additional file 5.** Quality assessment of the studies. Table showing the quality appraisal scores (two reviewers and average).

## Data Availability

The datasets used and/or analysed during the current study are included within the article and its additional files. Additional information is available from the corresponding author on reasonable request.

## Abbreviations

DHS: Demographic and Health Survey
LAM: Lactational Amenorrhoea Method
MMAT: Mixed Methods Appraisal Tool
PRISMA: Preferred Reporting Items for Systematic Reviews and Meta-Analyses
USAID: United States Agency for International Development
WHO: World Health Organization
