# Supplementary material for: Factors associated with short birth interval in low- and middle-income countries: a systematic review
Source: BMC Pregnancy Childbirth. 2020 Mar 12;20:156. doi: 10.1186/s12884-020-2852-z (PMC7069040; doi:10.1186/s12884-020-2852-z)
Supplement: Supplementary file 2 — Additional file 2. Search strategy. Key terms specific to each database, Boolean operators, and truncators. [file 12884_2020_2852_MOESM2_ESM.docx]

**Additional file 2 - Search strategy^[[1]](#footnote-1)^**

| **Pubmed** |
| --- |
| ((Developing Countries[Mesh:noexp] OR Africa[Mesh:noexp] OR Africa, Northern[Mesh:noexp] OR Africa South of the Sahara[Mesh:noexp] OR Africa, Central[Mesh:noexp] OR Africa, Eastern[Mesh:noexp] OR Africa, Southern[Mesh:noexp] OR Africa, Western[Mesh:noexp] OR Asia[Mesh:noexp] OR Asia, Central[Mesh:noexp] OR Asia, Southeastern[Mesh:noexp] OR Asia, Western[Mesh:noexp] OR Caribbean Region[Mesh:noexp] OR West Indies[Mesh:noexp] OR South America[Mesh:noexp] OR Latin America[Mesh:noexp] OR Central America[Mesh:noexp] OR Afghanistan[Mesh:noexp] OR Albania[Mesh:noexp] OR Algeria[Mesh:noexp] OR American Samoa[Mesh:noexp] OR Angola[Mesh:noexp] OR "Antigua and Barbuda"[Mesh:noexp] OR Argentina[Mesh:noexp] OR Armenia[Mesh:noexp] OR Azerbaijan[Mesh:noexp] OR Bahrain[Mesh:noexp] OR Bangladesh[Mesh:noexp] OR Barbados[Mesh:noexp] OR Benin[Mesh:noexp] OR Byelarus[Mesh:noexp] OR Belize[Mesh:noexp] OR Bhutan[Mesh:noexp] OR Bolivia[Mesh:noexp] OR Bosnia-Herzegovina[Mesh:noexp] OR Botswana[Mesh:noexp] OR Brazil[Mesh:noexp] OR Bulgaria[Mesh:noexp] OR Burkina Faso[Mesh:noexp] OR Burundi[Mesh:noexp] OR Cambodia[Mesh:noexp] OR Cameroon[Mesh:noexp] OR Cape Verde[Mesh:noexp] OR Central African Republic[Mesh:noexp] OR Chad[Mesh:noexp] OR Chile[Mesh:noexp] OR China[Mesh:noexp] OR Colombia[Mesh:noexp] OR Comoros[Mesh:noexp] OR Congo[Mesh:noexp] OR Costa Rica[Mesh:noexp] OR Cote d'Ivoire[Mesh:noexp] OR Croatia[Mesh:noexp] OR Cuba[Mesh:noexp] OR Cyprus[Mesh:noexp] OR Czechoslovakia[Mesh:noexp] OR Czech Republic[Mesh:noexp] OR Slovakia[Mesh:noexp] OR Djibouti[Mesh:noexp] OR "Democratic Republic of the Congo"[Mesh:noexp] OR Dominica[Mesh:noexp] OR Dominican Republic[Mesh:noexp] OR East Timor[Mesh:noexp] OR Ecuador[Mesh:noexp] OR Egypt[Mesh:noexp] OR El Salvador[Mesh:noexp] OR Eritrea[Mesh:noexp] OR Estonia[Mesh:noexp] OR Ethiopia[Mesh:noexp] OR Fiji[Mesh:noexp] OR Gabon[Mesh:noexp] OR Gambia[Mesh:noexp] OR "Georgia (Republic)"[Mesh:noexp] OR Ghana[Mesh:noexp] OR Greece[Mesh:noexp] OR Grenada[Mesh:noexp] OR Guatemala[Mesh:noexp] OR Guinea[Mesh:noexp] OR Guinea-Bissau[Mesh:noexp] OR Guam[Mesh:noexp] OR Guyana[Mesh:noexp] OR Haiti[Mesh:noexp] OR Honduras[Mesh:noexp] OR Hungary[Mesh:noexp] OR India[Mesh:noexp] OR Indonesia[Mesh:noexp] OR Iran[Mesh:noexp] OR Iraq[Mesh:noexp] OR Jamaica[Mesh:noexp] OR Jordan[Mesh:noexp] OR Kazakhstan[Mesh:noexp] OR Kenya[Mesh:noexp] OR Korea[Mesh:noexp] OR Kosovo[Mesh:noexp] OR Kyrgyzstan[Mesh:noexp] OR Laos[Mesh:noexp] OR Latvia[Mesh:noexp] OR Lebanon[Mesh:noexp] OR Lesotho[Mesh:noexp] OR Liberia[Mesh:noexp] OR Libya[Mesh:noexp] OR Lithuania[Mesh:noexp] OR Macedonia[Mesh:noexp] OR Madagascar[Mesh:noexp] OR Malaysia[Mesh:noexp] OR Malawi[Mesh:noexp] OR Mali[Mesh:noexp] OR Malta[Mesh:noexp] OR Mauritania[Mesh:noexp] OR Mauritius[Mesh:noexp] OR Mexico[Mesh:noexp] OR Micronesia[Mesh:noexp] OR Middle East[Mesh:noexp] OR Moldova[Mesh:noexp] OR Mongolia[Mesh:noexp] OR Montenegro[Mesh:noexp] OR Morocco[Mesh:noexp] OR Mozambique[Mesh:noexp] OR Myanmar[Mesh:noexp] OR Namibia[Mesh:noexp] OR Nepal[Mesh:noexp] OR Netherlands Antilles[Mesh:noexp] OR New Caledonia[Mesh:noexp] OR Nicaragua[Mesh:noexp] OR Niger[Mesh:noexp] OR Nigeria[Mesh:noexp] OR Oman[Mesh:noexp] OR Pakistan[Mesh:noexp] OR Palau[Mesh:noexp] OR Panama[Mesh:noexp] OR Papua New Guinea[Mesh:noexp] OR Paraguay[Mesh:noexp] OR Peru[Mesh:noexp] OR Philippines[Mesh:noexp] OR Poland[Mesh:noexp] OR Portugal[Mesh:noexp] OR Puerto Rico[Mesh:noexp] OR Romania[Mesh:noexp] OR Russia[Mesh:noexp] OR "Russia (Pre-1917)"[Mesh:noexp] OR Rwanda[Mesh:noexp] OR "Saint Kitts and Nevis"[Mesh:noexp] OR Saint Lucia[Mesh:noexp] OR "Saint Vincent and the Grenadines"[Mesh:noexp] OR Samoa[Mesh:noexp] OR Saudi Arabia[Mesh:noexp] OR Senegal[Mesh:noexp] OR Serbia[Mesh:noexp] OR Montenegro[Mesh:noexp] OR Seychelles[Mesh:noexp] OR Sierra Leone[Mesh:noexp] OR Slovenia[Mesh:noexp] OR Sri Lanka[Mesh:noexp] OR Somalia[Mesh:noexp] OR South Africa[Mesh:noexp] OR Sudan[Mesh:noexp] OR Suriname[Mesh:noexp] OR Swaziland[Mesh:noexp] OR Syria[Mesh:noexp] OR Tajikistan[Mesh:noexp] OR Tanzania[Mesh:noexp] OR Thailand[Mesh:noexp] OR Togo[Mesh:noexp] OR Tonga[Mesh:noexp] OR "Trinidad and Tobago"[Mesh:noexp] OR Tunisia[Mesh:noexp] OR Turkey[Mesh:noexp] OR Turkmenistan[Mesh:noexp] OR Uganda[Mesh:noexp] OR Ukraine[Mesh:noexp] OR Uruguay[Mesh:noexp] OR USSR[Mesh:noexp] OR Uzbekistan[Mesh:noexp] OR Vanuatu[Mesh:noexp] OR Venezuela[Mesh:noexp] OR Vietnam[Mesh:noexp] OR Yemen[Mesh:noexp] OR Yugoslavia[Mesh:noexp] OR Zambia[Mesh:noexp] OR Zimbabwe[Mesh:noexp]) OR (Macedonia[ot] OR Madagascar[ot] OR Malagasy Republic[ot] OR Malaysia[ot] OR Malaya[ot] OR Malay[ot] OR Sabah[ot] OR Sarawak[ot] OR Malawi[ot] OR Nyasaland[ot] OR Mali[ot] OR Malta[ot] OR Marshall Islands[ot] OR Mauritania[ot] OR Mauritius[ot] OR Agalega Islands[ot] OR Mexico[ot] OR Micronesia[ot] OR Middle East[ot] OR Moldova[ot] OR Moldovia[ot] OR Moldovian[ot] OR Mongolia[ot] OR Montenegro[ot] OR Morocco[ot] OR Ifni[ot] OR Mozambique[ot] OR Myanmar[ot] OR Myanma[ot] OR Burma[ot] OR Namibia[ot] OR Nepal[ot] OR Netherlands Antilles[ot] OR New Caledonia[ot] OR Nicaragua[ot] OR Niger[ot] OR Nigeria[ot] OR Northern Mariana Islands[ot] OR Oman[ot] OR Muscat[ot] OR Pakistan[ot] OR Palau[ot] OR Palestine[ot] OR Panama[ot] OR Paraguay[ot] OR Peru[ot] OR Philippines[ot] OR Philipines[ot] OR Phillipines[ot] OR Phillippines[ot] OR Poland[ot] OR Portugal[ot] OR Puerto Rico[ot] OR Romania[ot] OR Rumania[ot] OR Roumania[ot] OR Russia[ot] OR Russian[ot] OR Rwanda[ot] OR Ruanda[ot] OR Saint Kitts[ot] OR St Kitts[ot] OR Nevis[ot] OR Saint Lucia[ot] OR St Lucia[ot] OR Saint Vincent[ot] OR St Vincent[ot] OR Grenadines[ot] OR Samoa[ot] OR Samoan Islands[ot] OR Navigator Island[ot] OR Navigator Islands[ot] OR Sao Tome[ot] OR Saudi Arabia[ot] OR Senegal[ot] OR Serbia[ot] OR Montenegro[ot] OR Seychelles[ot] OR Sierra Leone[ot] OR Slovenia[ot] OR Sri Lanka[ot] OR Ceylon[ot] OR Solomon Islands[ot] OR Somalia[ot] OR Sudan[ot] OR Suriname[ot] OR Surinam[ot] OR Swaziland[ot] OR Syria[ot] OR Tajikistan[ot] OR Tadzhikistan[ot] OR Tadjikistan[ot] OR Tadzhik[ot] OR Tanzania[ot] OR Thailand[ot] OR Togo[ot] OR Togolese Republic[ot] OR Tonga[ot] OR Trinidad[ot] OR Tobago[ot] OR Tunisia[ot] OR Turkey[ot] OR Turkmenistan[ot] OR Turkmen[ot] OR Uganda[ot] OR Ukraine[ot] OR Uruguay[ot] OR USSR[ot] OR Soviet Union[ot] OR Union of Soviet Socialist Republics[ot] OR Uzbekistan[ot] OR Uzbek OR Vanuatu[ot] OR New Hebrides[ot] OR Venezuela[ot] OR Vietnam[ot] OR Viet Nam[ot] OR West Bank[ot] OR Yemen[ot] OR Yugoslavia[ot] OR Zambia[ot] OR Zimbabwe[ot] OR Rhodesia[ot]) OR (Africa[ot] OR Asia[ot] OR Caribbean[ot] OR West Indies[ot] OR South America[ot] OR Latin America[ot] OR Central America[ot] OR Afghanistan[ot] OR Albania[ot] OR Algeria[ot] OR Angola[ot] OR Antigua[ot] OR Barbuda[ot] OR Argentina[ot] OR Armenia[ot] OR Armenian[ot] OR Aruba[ot] OR Azerbaijan[ot] OR Bahrain[ot] OR Bangladesh[ot] OR Barbados[ot] OR Benin[ot] OR Byelarus[ot] OR Byelorussian[ot] OR Belarus[ot] OR Belorussian[ot] OR Belorussia[ot] OR Belize[ot] OR Bhutan[ot] OR Bolivia[ot] OR Bosnia[ot] OR Herzegovina[ot] OR Hercegovina[ot] OR Botswana[ot] OR Brasil[ot] OR Brazil[ot] OR Bulgaria[ot] OR Burkina Faso[ot] OR Burkina Fasso[ot] OR Upper Volta[ot] OR Burundi[ot] OR Urundi[ot] OR Cambodia[ot] OR Khmer Republic[ot] OR Kampuchea[ot] OR Cameroon[ot] OR Cameroons[ot] OR Cameron[ot] OR Camerons[ot] OR Cape Verde[ot] OR Central African Republic[ot] OR Chad[ot] OR Chile[ot] OR China[ot] OR Colombia[ot] OR Comoros[ot] OR Comoro Islands[ot] OR Comores[ot] OR Mayotte[ot] OR Congo[ot] OR Zaire[ot] OR Costa Rica[ot] OR Cote d'Ivoire[ot] OR Ivory Coast[ot] OR Croatia[ot] OR Cuba[ot] OR Cyprus[ot] OR Czechoslovakia[ot] OR Czech Republic[ot] OR Slovakia[ot] OR Slovak Republic[ot] OR Djibouti[ot] OR French Somaliland[ot] OR Dominica[ot] OR Dominican Republic[ot] OR East Timor[ot] OR East Timur[ot] OR Timor Leste[ot] OR Ecuador[ot] OR Egypt[ot] OR United Arab Republic[ot] OR El Salvador[ot] OR Eritrea[ot] OR Estonia[ot] OR Ethiopia[ot] OR Fiji[ot] OR Gabon[ot] OR Gabonese Republic[ot] OR Gambia[ot] OR Gaza[ot] OR "Georgia Republic"[ot] OR "Georgian Republic"[ot] OR Ghana[ot] OR Gold Coast[ot] OR Greece[ot] OR Grenada[ot] OR Guatemala[ot] OR Guinea[ot] OR Guam[ot] OR Guiana[ot] OR Guyana[ot] OR Haiti[ot] OR Honduras[ot] OR Hungary[ot] OR India[ot] OR Maldives[ot] OR Indonesia[ot] OR Iran[ot] OR Iraq[ot] OR Isle of Man[ot] OR Jamaica[ot] OR Jordan[ot] OR Kazakhstan[ot] OR Kazakh[ot] OR Kenya[ot] OR Kiribati[ot] OR Korea[ot] OR Kosovo[ot] OR Kyrgyzstan[ot] OR Kirghizia[ot] OR Kyrgyz Republic[ot] OR Kirghiz[ot] OR Kirgizstan[ot] OR "Lao PDR"[ot] OR Laos[ot] OR Latvia[ot] OR Lebanon[ot] OR Lesotho[ot] OR Basutoland[ot] OR Liberia[ot] OR Libya[ot] OR Lithuania[ot]) OR (Macedonia[tiab] OR Madagascar[tiab] OR Malagasy Republic[tiab] OR Malaysia[tiab] OR Malaya[tiab] OR Malay[tiab] OR Sabah[tiab] OR Sarawak[tiab] OR Malawi[tiab] OR Nyasaland[tiab] OR Mali[tiab] OR Malta[tiab] OR Marshall Islands[tiab] OR Mauritania[tiab] OR Mauritius[tiab] OR Agalega Islands[tiab] OR Mexico[tiab] OR Micronesia[tiab] OR Middle East[tiab] OR Moldova[tiab] OR Moldovia[tiab] OR Moldovian[tiab] OR Mongolia[tiab] OR Montenegro[tiab] OR Morocco[tiab] OR Ifni[tiab] OR Mozambique[tiab] OR Myanmar[tiab] OR Myanma[tiab] OR Burma[tiab] OR Namibia[tiab] OR Nepal[tiab] OR Netherlands Antilles[tiab] OR New Caledonia[tiab] OR Nicaragua[tiab] OR Niger[tiab] OR Nigeria[tiab] OR Northern Mariana Islands[tiab] OR Oman[tiab] OR Muscat[tiab] OR Pakistan[tiab] OR Palau[tiab] OR Palestine[tiab] OR Panama[tiab] OR Paraguay[tiab] OR Peru[tiab] OR Philippines[tiab] OR Philipines[tiab] OR Phillipines[tiab] OR Phillippines[tiab] OR Poland[tiab] OR Portugal[tiab] OR Puerto Rico[tiab] OR Romania[tiab] OR Rumania[tiab] OR Roumania[tiab] OR Russia[tiab] OR Russian[tiab] OR Rwanda[tiab] OR Ruanda[tiab] OR Saint Kitts[tiab] OR St Kitts[tiab] OR Nevis[tiab] OR Saint Lucia[tiab] OR St Lucia[tiab] OR Saint Vincent[tiab] OR St Vincent[tiab] OR Grenadines[tiab] OR Samoa[tiab] OR Samoan Islands[tiab] OR Navigator Island[tiab] OR Navigator Islands[tiab] OR Sao Tome[tiab] OR Saudi Arabia[tiab] OR Senegal[tiab] OR Serbia[tiab] OR Montenegro[tiab] OR Seychelles[tiab] OR Sierra Leone[tiab] OR Slovenia[tiab] OR Sri Lanka[tiab] OR Ceylon[tiab] OR Solomon Islands[tiab] OR Somalia[tiab] OR Sudan[tiab] OR Suriname[tiab] OR Surinam[tiab] OR Swaziland[tiab] OR Syria[tiab] OR Tajikistan[tiab] OR Tadzhikistan[tiab] OR Tadjikistan[tiab] OR Tadzhik[tiab] OR Tanzania[tiab] OR Thailand[tiab] OR Togo[tiab] OR Togolese Republic[tiab] OR Tonga[tiab] OR Trinidad[tiab] OR Tobago[tiab] OR Tunisia[tiab] OR Turkey[tiab] OR Turkmenistan[tiab] OR Turkmen[tiab] OR Uganda[tiab] OR Ukraine[tiab] OR Uruguay[tiab] OR USSR[tiab] OR Soviet Union[tiab] OR Union of Soviet Socialist Republics[tiab] OR Uzbekistan[tiab] OR Uzbek OR Vanuatu[tiab] OR New Hebrides[tiab] OR Venezuela[tiab] OR Vietnam[tiab] OR Viet Nam[tiab] OR West Bank[tiab] OR Yemen[tiab] OR Yugoslavia[tiab] OR Zambia[tiab] OR Zimbabwe[tiab] OR Rhodesia[tiab]) OR (Africa[tiab] OR Asia[tiab] OR Caribbean[tiab] OR West Indies[tiab] OR South America[tiab] OR Latin America[tiab] OR Central America[tiab] OR Afghanistan[tiab] OR Albania[tiab] OR Algeria[tiab] OR Angola[tiab] OR Antigua[tiab] OR Barbuda[tiab] OR Argentina[tiab] OR Armenia[tiab] OR Armenian[tiab] OR Aruba[tiab] OR Azerbaijan[tiab] OR Bahrain[tiab] OR Bangladesh[tiab] OR Barbados[tiab] OR Benin[tiab] OR Byelarus[tiab] OR Byelorussian[tiab] OR Belarus[tiab] OR Belorussian[tiab] OR Belorussia[tiab] OR Belize[tiab] OR Bhutan[tiab] OR Bolivia[tiab] OR Bosnia[tiab] OR Herzegovina[tiab] OR Hercegovina[tiab] OR Botswana[tiab] OR Brasil[tiab] OR Brazil[tiab] OR Bulgaria[tiab] OR Burkina Faso[tiab] OR Burkina Fasso[tiab] OR Upper Volta[tiab] OR Burundi[tiab] OR Urundi[tiab] OR Cambodia[tiab] OR Khmer Republic[tiab] OR Kampuchea[tiab] OR Cameroon[tiab] OR Cameroons[tiab] OR Cameron[tiab] OR Camerons[tiab] OR Cape Verde[tiab] OR Central African Republic[tiab] OR Chad[tiab] OR Chile[tiab] OR China[tiab] OR Colombia[tiab] OR Comoros[tiab] OR Comoro Islands[tiab] OR Comores[tiab] OR Mayotte[tiab] OR Congo[tiab] OR Zaire[tiab] OR Costa Rica[tiab] OR Cote d'Ivoire[tiab] OR Ivory Coast[tiab] OR Croatia[tiab] OR Cuba[tiab] OR Cyprus[tiab] OR Czechoslovakia[tiab] OR Czech Republic[tiab] OR Slovakia[tiab] OR Slovak Republic[tiab] OR Djibouti[tiab] OR French Somaliland[tiab] OR Dominica[tiab] OR Dominican Republic[tiab] OR East Timor[tiab] OR East Timur[tiab] OR Timor Leste[tiab] OR Ecuador[tiab] OR Egypt[tiab] OR United Arab Republic[tiab] OR El Salvador[tiab] OR Eritrea[tiab] OR Estonia[tiab] OR Ethiopia[tiab] OR Fiji[tiab] OR Gabon[tiab] OR Gabonese Republic[tiab] OR Gambia[tiab] OR Gaza[tiab] OR Georgia Republic[tiab] OR Georgian Republic[tiab] OR Ghana[tiab] OR Gold Coast[tiab] OR Greece[tiab] OR Grenada[tiab] OR Guatemala[tiab] OR Guinea[tiab] OR Guam[tiab] OR Guiana[tiab] OR Guyana[tiab] OR Haiti[tiab] OR Honduras[tiab] OR Hungary[tiab] OR India[tiab] OR Maldives[tiab] OR Indonesia[tiab] OR Iran[tiab] OR Iraq[tiab] OR Isle of Man[tiab] OR Jamaica[tiab] OR Jordan[tiab] OR Kazakhstan[tiab] OR Kazakh[tiab] OR Kenya[tiab] OR Kiribati[tiab] OR Korea[tiab] OR Kosovo[tiab] OR Kyrgyzstan[tiab] OR Kirghizia[tiab] OR Kyrgyz Republic[tiab] OR Kirghiz[tiab] OR Kirgizstan[tiab] OR "Lao PDR"[tiab] OR Laos[tiab] OR Latvia[tiab] OR Lebanon[tiab] OR Lesotho[tiab] OR Basutoland[tiab] OR Liberia[tiab] OR Libya[tiab] OR Lithuania[tiab]) OR (Macedonia[pl] OR Madagascar[pl] OR Malagasy Republic[pl] OR Malaysia[pl] OR Malaya[pl] OR Malay[pl] OR Sabah[pl] OR Sarawak[pl] OR Malawi[pl] OR Nyasaland[pl] OR Mali[pl] OR Malta[pl] OR Marshall Islands[pl] OR Mauritania[pl] OR Mauritius[pl] OR Agalega Islands[pl] OR Mexico[pl] OR Micronesia[pl] OR Middle East[pl] OR Moldova[pl] OR Moldovia[pl] OR Moldovian[pl] OR Mongolia[pl] OR Montenegro[pl] OR Morocco[pl] OR Ifni[pl] OR Mozambique[pl] OR Myanmar[pl] OR Myanma[pl] OR Burma[pl] OR Namibia[pl] OR Nepal[pl] OR Netherlands Antilles[pl] OR New Caledonia[pl] OR Nicaragua[pl] OR Niger[pl] OR Nigeria[pl] OR Northern Mariana Islands[pl] OR Oman[pl] OR Muscat[pl] OR Pakistan[pl] OR Palau[pl] OR Palestine[pl] OR Panama[pl] OR Paraguay[pl] OR Peru[pl] OR Philippines[pl] OR Philipines[pl] OR Phillipines[pl] OR Phillippines[pl] OR Poland[pl] OR Portugal[pl] OR Puerto Rico[pl] OR Romania[pl] OR Rumania[pl] OR Roumania[pl] OR Russia[pl] OR Russian[pl] OR Rwanda[pl] OR Ruanda[pl] OR Saint Kitts[pl] OR St Kitts[pl] OR Nevis[pl] OR Saint Lucia[pl] OR St Lucia[pl] OR Saint Vincent[pl] OR St Vincent[pl] OR Grenadines[pl] OR Samoa[pl] OR Samoan Islands[pl] OR Navigator Island[pl] OR Navigator Islands[pl] OR Sao Tome[pl] OR Saudi Arabia[pl] OR Senegal[pl] OR Serbia[pl] OR Montenegro[pl] OR Seychelles[pl] OR Sierra Leone[pl] OR Slovenia[pl] OR Sri Lanka[pl] OR Ceylon[pl] OR Solomon Islands[pl] OR Somalia[pl] OR South Africa[pl] OR Sudan[pl] OR Suriname[pl] OR Surinam[pl] OR Swaziland[pl] OR Syria[pl] OR Tajikistan[pl] OR Tadzhikistan[pl] OR Tadjikistan[pl] OR Tadzhik[pl] OR Tanzania[pl] OR Thailand[pl] OR Togo[pl] OR Togolese Republic[pl] OR Tonga[pl] OR Trinidad[pl] OR Tobago[pl] OR Tunisia[pl] OR Turkey[pl] OR Turkmenistan[pl] OR Turkmen[pl] OR Uganda[pl] OR Ukraine[pl] OR Uruguay[pl] OR USSR[pl] OR Soviet Union[pl] OR Union of Soviet Socialist Republics[pl] OR Uzbekistan[pl] OR Uzbek OR Vanuatu[pl] OR New Hebrides[pl] OR Venezuela[pl] OR Vietnam[pl] OR Viet Nam[pl] OR West Bank[pl] OR Yemen[pl] OR Yugoslavia[pl] OR Zambia[pl] OR Zimbabwe[pl] OR Rhodesia[pl]) OR (Africa[pl] OR Asia[pl] OR Caribbean[pl] OR West Indies[pl] OR South America[pl] OR Latin America[pl] OR Central America[pl] OR Afghanistan[pl] OR Albania[pl] OR Algeria[pl] OR Angola[pl] OR Antigua[pl] OR Barbuda[pl] OR Argentina[pl] OR Armenia[pl] OR Armenian[pl] OR Aruba[pl] OR Azerbaijan[pl] OR Bahrain[pl] OR Bangladesh[pl] OR Barbados[pl] OR Benin[pl] OR Byelarus[pl] OR Byelorussian[pl] OR Belarus[pl] OR Belorussian[pl] OR Belorussia[pl] OR Belize[pl] OR Bhutan[pl] OR Bolivia[pl] OR Bosnia[pl] OR Herzegovina[pl] OR Hercegovina[pl] OR Botswana[pl] OR Brasil[pl] OR Brazil[pl] OR Bulgaria[pl] OR Burkina Faso[pl] OR Burkina Fasso[pl] OR Upper Volta[pl] OR Burundi[pl] OR Urundi[pl] OR Cambodia[pl] OR Khmer Republic[pl] OR Kampuchea[pl] OR Cameroon[pl] OR Cameroons[pl] OR Cameron[pl] OR Camerons[pl] OR Cape Verde[pl] OR Central African Republic[pl] OR Chad[pl] OR Chile[pl] OR China[pl] OR Colombia[pl] OR Comoros[pl] OR Comoro Islands[pl] OR Comores[pl] OR Mayotte[pl] OR Congo[pl] OR Zaire[pl] OR Costa Rica[pl] OR Cote d'Ivoire[pl] OR Ivory Coast[pl] OR Croatia[pl] OR Cuba[pl] OR Cyprus[pl] OR Czechoslovakia[pl] OR Czech Republic[pl] OR Slovakia[pl] OR Slovak Republic[pl] OR Djibouti[pl] OR French Somaliland[pl] OR Dominica[pl] OR Dominican Republic[pl] OR East Timor[pl] OR East Timur[pl] OR Timor Leste[pl] OR Ecuador[pl] OR Egypt[pl] OR United Arab Republic[pl] OR El Salvador[pl] OR Eritrea[pl] OR Estonia[pl] OR Ethiopia[pl] OR Fiji[pl] OR Gabon[pl] OR Gabonese Republic[pl] OR Gambia[pl] OR Gaza[pl] OR Georgia Republic[pl] OR Georgian Republic[pl] OR Ghana[pl] OR Gold Coast[pl] OR Greece[pl] OR Grenada[pl] OR Guatemala[pl] OR Guinea[pl] OR Guam[pl] OR Guiana[pl] OR Guyana[pl] OR Haiti[pl] OR Honduras[pl] OR Hungary[pl] OR India[pl] OR Maldives[pl] OR Indonesia[pl] OR Iran[pl] OR Iraq[pl] OR Isle of Man[pl] OR Jamaica[pl] OR Jordan[pl] OR Kazakhstan[pl] OR Kazakh[pl] OR Kenya[pl] OR Kiribati[pl] OR Korea[pl] OR Kosovo[pl] OR Kyrgyzstan[pl] OR Kirghizia[pl] OR Kyrgyz Republic[pl] OR Kirghiz[pl] OR Kirgizstan[pl] OR "Lao PDR"[pl] OR Laos[pl] OR Latvia[pl] OR Lebanon[pl] OR Lesotho[pl] OR Basutoland[pl] OR Liberia[pl] OR Libya[pl] OR Lithuania[pl]) OR ("developing country"[ot] OR "developing countries"[ot] OR "developing nation"[ot] OR "developing nations"[ot] OR "developing population"[ot] OR "developing populations"[ot] OR "developing world"[ot] OR "less developed country"[ot] OR "less developed countries"[ot] OR "less developed nation"[ot] OR "less developed nations"[ot] OR "less developed population"[ot] OR "less developed populations"[ot] OR "less developed world"[ot] OR "lesser developed country"[ot] OR "lesser developed countries"[ot] OR "lesser developed nation"[ot] OR "lesser developed nations"[ot] OR "lesser developed population"[ot] OR "lesser developed populations"[ot] OR "lesser developed world"[ot] OR "under developed country"[ot] OR "under developed countries"[ot] OR "under developed nation"[ot] OR "under developed nations"[ot] OR "under developed population"[ot] OR "under developed populations"[ot] OR "under developed world"[ot] OR "underdeveloped country"[ot] OR "underdeveloped countries"[ot] OR "underdeveloped nation"[ot] OR "underdeveloped nations"[ot] OR "underdeveloped population"[ot] OR "underdeveloped populations"[ot] OR "underdeveloped world"[ot] OR "middle income country"[ot] OR "middle income countries"[ot] OR "middle income nation"[ot] OR "middle income nations"[ot] OR "middle income population"[ot] OR "middle income populations"[ot] OR "low income country"[ot] OR "low income countries"[ot] OR "low income nation"[ot] OR "low income nations"[ot] OR "low income population"[ot] OR "low income populations"[ot] OR "lower income country"[ot] OR "lower income countries"[ot] OR "lower income nation"[ot] OR "lower income nations"[ot] OR "lower income population"[ot] OR "lower income populations"[ot] OR "underserved country"[ot] OR "underserved countries"[ot] OR "underserved nation"[ot] OR "underserved nations"[ot] OR "underserved population"[ot] OR "underserved populations"[ot] OR "underserved world"[ot] OR "under served country"[ot] OR "under served countries"[ot] OR "under served nation"[ot] OR "under served nations"[ot] OR "under served population"[ot] OR "under served populations"[ot] OR "under served world"[ot] OR "deprived country"[ot] OR "deprived countries"[ot] OR "deprived nation"[ot] OR "deprived nations"[ot] OR "deprived population"[ot] OR "deprived populations"[ot] OR "deprived world"[ot] OR "poor country"[ot] OR "poor countries"[ot] OR "poor nation"[ot] OR "poor nations"[ot] OR "poor population"[ot] OR "poor populations"[ot] OR "poor world"[ot] OR "poorer country"[ot] OR "poorer countries"[ot] OR "poorer nation"[ot] OR "poorer nations"[ot] OR "poorer population"[ot] OR "poorer populations"[ot] OR "poorer world"[ot] OR "developing economy"[ot] OR "developing economies"[ot] OR "less developed economy"[ot] OR "less developed economies"[ot] OR "lesser developed economy"[ot] OR "lesser developed economies"[ot] OR "under developed economy"[ot] OR "under developed economies"[ot] OR "underdeveloped economy"[ot] OR "underdeveloped economies"[ot] OR "middle income economy"[ot] OR "middle income economies"[ot] OR "low income economy"[ot] OR "low income economies"[ot] OR "lower income economy"[ot] OR "lower income economies"[ot] OR "low gdp"[ot] OR "low gnp"[ot] OR "low gross domestic"[ot] OR "low gross national"[ot] OR "lower gdp"[ot] OR "lower gnp"[ot] OR "lower gross domestic"[ot] OR "lower gross national"[ot] OR lmic[ot] OR lmics[ot] OR "third world"[ot] OR "lami country"[ot] OR "lami countries"[ot] OR "transitional country"[ot] OR "transitional countries"[ot]) OR ("developing country"[tiab] OR "developing countries"[tiab] OR "developing nation"[tiab] OR "developing nations"[tiab] OR "developing population"[tiab] OR "developing populations"[tiab] OR "developing world"[tiab] OR "less developed country"[tiab] OR "less developed countries"[tiab] OR "less developed nation"[tiab] OR "less developed nations"[tiab] OR "less developed population"[tiab] OR "less developed populations"[tiab] OR "less developed world"[tiab] OR "lesser developed country"[tiab] OR "lesser developed countries"[tiab] OR "lesser developed nation"[tiab] OR "lesser developed nations"[tiab] OR "lesser developed population"[tiab] OR "lesser developed populations"[tiab] OR "lesser developed world"[tiab] OR "under developed country"[tiab] OR "under developed countries"[tiab] OR "under developed nation"[tiab] OR "under developed nations"[tiab] OR "under developed population"[tiab] OR "under developed populations"[tiab] OR "under developed world"[tiab] OR "underdeveloped country"[tiab] OR "underdeveloped countries"[tiab] OR "underdeveloped nation"[tiab] OR "underdeveloped nations"[tiab] OR "underdeveloped population"[tiab] OR "underdeveloped populations"[tiab] OR "underdeveloped world"[tiab] OR "middle income country"[tiab] OR "middle income countries"[tiab] OR "middle income nation"[tiab] OR "middle income nations"[tiab] OR "middle income population"[tiab] OR "middle income populations"[tiab] OR "low income country"[tiab] OR "low income countries"[tiab] OR "low income nation"[tiab] OR "low income nations"[tiab] OR "low income population"[tiab] OR "low income populations"[tiab] OR "lower income country"[tiab] OR "lower income countries"[tiab] OR "lower income nation"[tiab] OR "lower income nations"[tiab] OR "lower income population"[tiab] OR "lower income populations"[tiab] OR "underserved country"[tiab] OR "underserved countries"[tiab] OR "underserved nation"[tiab] OR "underserved nations"[tiab] OR "underserved population"[tiab] OR "underserved populations"[tiab] OR "underserved world"[tiab] OR "under served country"[tiab] OR "under served countries"[tiab] OR "under served nation"[tiab] OR "under served nations"[tiab] OR "under served population"[tiab] OR "under served populations"[tiab] OR "under served world"[tiab] OR "deprived country"[tiab] OR "deprived countries"[tiab] OR "deprived nation"[tiab] OR "deprived nations"[tiab] OR "deprived population"[tiab] OR "deprived populations"[tiab] OR "deprived world"[tiab] OR "poor country"[tiab] OR "poor countries"[tiab] OR "poor nation"[tiab] OR "poor nations"[tiab] OR "poor population"[tiab] OR "poor populations"[tiab] OR "poor world"[tiab] OR "poorer country"[tiab] OR "poorer countries"[tiab] OR "poorer nation"[tiab] OR "poorer nations"[tiab] OR "poorer population"[tiab] OR "poorer populations"[tiab] OR "poorer world"[tiab] OR "developing economy"[tiab] OR "developing economies"[tiab] OR "less developed economy"[tiab] OR "less developed economies"[tiab] OR "lesser developed economy"[tiab] OR "lesser developed economies"[tiab] OR "under developed economy"[tiab] OR "under developed economies"[tiab] OR "underdeveloped economy"[tiab] OR "underdeveloped economies"[tiab] OR "middle income economy"[tiab] OR "middle income economies"[tiab] OR "low income economy"[tiab] OR "low income economies"[tiab] OR "lower income economy"[tiab] OR "lower income economies"[tiab] OR "low gdp"[tiab] OR "low gnp"[tiab] OR "low gross domestic"[tiab] OR "low gross national"[tiab] OR "lower gdp"[tiab] OR "lower gnp"[tiab] OR "lower gross domestic"[tiab] OR "lower gross national"[tiab] OR lmic[tiab] OR lmics[tiab] OR "third world"[tiab] OR "lami country"[tiab] OR "lami countries"[tiab] OR "transitional country"[tiab] OR "transitional countries"[tiab])) AND and AND ("Age Factors"[Mesh] OR "Precipitating Factors"[Mesh] OR "Risk Factors"[Mesh] OR "Sex Factors"[Mesh] OR "Socioeconomic Factors"[Mesh] OR "Residence Characteristics"[Mesh] OR "Social Conditions"[Mesh] OR "Social Environment"[Mesh] OR "Social Isolation"[Mesh] OR "Social Change"[Mesh] OR "Social Marginalization"[Mesh] OR "Social Norms"[Mesh] OR "Socialization"[Mesh] OR "Sociological Factors"[Mesh] OR "Environmental Exposure"[Mesh] OR Time Factors[Mesh] OR determinant*[Text Word] OR predictor*[Text Word]) AND and AND ("Birth Intervals"[Mesh] OR Birth Spacing*[Text Word] OR Pregnancy Interval*[Text Word] OR birth interval*[Text Word] OR interpregnancy interval*[Text Word] OR interbirth interval*[Text Word] OR rapid repeat pregnancy*[Text Word] OR pregnancy spacing*[Text Word] OR espacement des naissance*[Text Word] OR Repeat teenage pregnancy[Text Word] OR Repeat teenage pregnancies[Text Word] OR Child Spacing*[Text Word]) |
| **Embase** |
| 1. (Birth Intervals or Birth Spacing* or Pregnancy Interval* or birth interval* or interpregnancy interval* or interbirth interval* or rapid repeat pregnancy* or pregnancy spacing* or espacement des naissance* or Repeat teenage pregnancy or Repeat teenage pregnancies or Child Spacing*).mp.  2. Developing Country.sh.  3. (Africa or Asia or Caribbean or West Indies or South America or Latin America or Central America).hw,ti,ab,cp.  4. (Afghanistan or Albania or Algeria or Angola or Antigua or Barbuda or Argentina or Armenia or Armenian or Aruba or Azerbaijan or Bahrain or Bangladesh or Barbados or Benin or Byelarus or Byelorussian or Belarus or Belorussian or Belorussia or Belize or Bhutan or Bolivia or Bosnia or Herzegovina or Hercegovina or Botswana or Brasil or Brazil or Bulgaria or Burkina Faso or Burkina Fasso or Upper Volta or Burundi or Urundi or Cambodia or Khmer Republic or Kampuchea or Cameroon or Cameroons or Cameron or Camerons or Cape Verde or Central African Republic or Chad or Chile or China or Colombia or Comoros or Comoro Islands or Comores or Mayotte or Congo or Zaire or Costa Rica or Cote d'Ivoire or Ivory Coast or Croatia or Cuba or Cyprus or Czechoslovakia or Czech Republic or Slovakia or Slovak Republic or Djibouti or French Somaliland or Dominica or Dominican Republic or East Timor or East Timur or Timor Leste or Ecuador or Egypt or United Arab Republic or El Salvador or Eritrea or Estonia or Ethiopia or Fiji or Gabon or Gabonese Republic or Gambia or Gaza or Georgia Republic or Georgian Republic or Ghana or Gold Coast or Greece or Grenada or Guatemala or Guinea or Guam or Guiana or Guyana or Haiti or Honduras or Hungary or India or Maldives or Indonesia or Iran or Iraq or Isle of Man or Jamaica or Jordan or Kazakhstan or Kazakh or Kenya or Kiribati or Korea or Kosovo or Kyrgyzstan or Kirghizia or Kyrgyz Republic or Kirghiz or Kirgizstan or Lao PDR or Laos or Latvia or Lebanon or Lesotho or Basutoland or Liberia or Libya or Lithuania or Macedonia or Madagascar or Malagasy Republic or Malaysia or Malaya or Malay or Sabah or Sarawak or Malawi or Nyasaland or Mali or Malta or Marshall Islands or Mauritania or Mauritius or Agalega Islands or Mexico or Micronesia or Middle East or Moldova or Moldovia or Moldovian or Mongolia or Montenegro or Morocco or Ifni or Mozambique or Myanmar or Myanma or Burma or Namibia or Nepal or Netherlands Antilles or New Caledonia or Nicaragua or Niger or Nigeria or Northern Mariana Islands or Oman or Muscat or Pakistan or Palau or Palestine or Panama or Paraguay or Peru or Philippines or Philipines or Phillipines or Phillippines or Poland or Portugal or Puerto Rico or Romania or Rumania or Roumania or Russia or Russian or Rwanda or Ruanda or Saint Kitts or St Kitts or Nevis or Saint Lucia or St Lucia or Saint Vincent or St Vincent or Grenadines or Samoa or Samoan Islands or Navigator Island or Navigator Islands or Sao Tome or Saudi Arabia or Senegal or Serbia or Montenegro or Seychelles or Sierra Leone or Slovenia or Sri Lanka or Ceylon or Solomon Islands or Somalia or South Africa or Sudan or Suriname or Surinam or Swaziland or Syria or Tajikistan or Tadzhikistan or Tadjikistan or Tadzhik or Tanzania or Thailand or Togo or Togolese Republic or Tonga or Trinidad or Tobago or Tunisia or Turkey or Turkmenistan or Turkmen or Uganda or Ukraine or Uruguay or USSR or Soviet Union or Union of Soviet Socialist Republics or Uzbekistan or Uzbek or Vanuatu or New Hebrides or Venezuela or Vietnam or Viet Nam or West Bank or Yemen or Yugoslavia or Zambia or Zimbabwe or Rhodesia).hw,ti,ab,cp.  5. ((developing or less* developed or under developed or underdeveloped or middle income or low* income or underserved or under served or deprived or poor*) adj (countr* or nation? or population? or world)).ti,ab.  6. ((developing or less* developed or under developed or underdeveloped or middle income or low* income) adj (economy or economies)).ti,ab.  7. (low* adj (gdp or gnp or gross domestic or gross national)).ti,ab.  8. (low adj3 middle adj3 countr*).ti,ab.  9. (lmic or lmics or third world or lami countr*).ti,ab.  10. transitional countr*.ti,ab.  11. or/2-10  12. age/ or Precipitating Factors.mp. or *risk factor/ or *sex factor/ or *socioeconomics/ or demography/ or *social status/ or *social environment/ or *social isolation/ or *social change/ or *social exclusion/ or *social norm/ or *socialization/ or *"social aspects and related phenomena"/ or *environmental exposure/ or *time factor/ or determinant*/ or predictor*/  13. 1 and 11 and 12 |
| **Lilacs** |
| "Birth Intervals" or "Birth Spacing" or "Birth Spacings" or "Pregnancy Interval" or "Pregnancy Intervals" or "birth interval" or "interpregnancy interval" or "interpregnancy intervals" or "interbirth interval" or "interbirth intervals" or "rapid repeat pregnancy" or "pregnancy spacing" or "pregnancy spacings" or "Repeat teenage pregnancy" or "Repeat teenage pregnancies" or "Child Spacing" or "Child Spacings" |
| **Popline** |
| (AGE FACTORS OR RISK FACTORS OR SEX FACTORS OR SOCIOECONOMIC FACTORS OR RESIDENCE OR CHARACTERISTICS OR SOCIAL CHANGE OR TIME FACTORS OR DEMOGRAPHIC FACTORS OR ECONOMIC FACTORS OR GEOGRAPHIC FACTORS OR SOCIOCULTURAL FACTORS OR PSYCHOSOCIAL FACTORS) AND (BIRTH INTERVALS OR BIRTH SPACING OR PREGNANCY INTERVALS OR FIRST BIRTH INTERVALS OR FIRST PREGNANCY INTERVALS OR OPEN LIVE-BIRTH INTERVALS) AND (AFRICA OR SOUTH AMERICA OR CENTRAL AMERICA OR ASIA) |

1. For all the databases the date last searched was 24/10/2018 [↑](#footnote-ref-1)
