# Supplementary material for: Factors associated with short birth interval in low- and middle-income countries: a systematic review
Source: BMC Pregnancy Childbirth. 2020 Mar 12;20:156. doi: 10.1186/s12884-020-2852-z (PMC7069040; doi:10.1186/s12884-020-2852-z)
Supplement: Supplementary file 3 — Additional file 3. Full references of the records included in our study. List showing the full references of the documents included in our study. [file 12884_2020_2852_MOESM3_ESM.docx]

**Additional file 3. Full references of the records included in our study**

1. Todd CS, Isley MM, Ahmadzai M, Azfar P, Atiqzai F, Smith JM, et al. Cross-sectional analysis of factors associated with prior contraceptive use among hospitalized obstetric patients in Kabul, Afghanistan. Contraception. 2008;78:249–56.

2. Baqui AH, Ahmed S, Begum N, Khanam R, Mohan D, Harrison M, et al. Impact of integrating a postpartum family planning program into a community-based maternal and newborn health program on birth spacing and preterm birth in rural Bangladesh. J Glob Health. 2018;8:20406.

3. de Jonge HCC, Azad K, Seward N, Kuddus A, Shaha S, Beard J, et al. Determinants and consequences of short birth interval in rural Bangladesh: a cross-sectional study. BMC Pregnancy Childbirth. 2014;14:427.

4. França Júnior I, Wagner MB, Ibañez RN, Giugliani ERJ. Análise do espaçamento intergestacional em uma vila periférica de Porto Alegre. Rev HCPA Fac Med Univ Fed Rio Gd do Sul. 1985;5:21–4.

5. Ngianga-Bakwin K, Stones RW. Birth intervals and injectable contraception in sub-Saharan Africa. Contraception. 2005;71:353–6.

6. Chirwa TF, Mantempa JN, Kinziunga FL, Kandala JD, Kandala N-B. An exploratory spatial analysis of geographical inequalities of birth intervals among young women in the Democratic Republic of Congo (DRC): a cross-sectional study. BMC Pregnancy Childbirth. 2014;14:271.

7. Begna Z, Assegid S, Kassahun W, Gerbaba M. Determinants of inter birth interval among married women living in rural pastoral communities of southern Ethiopia: a case control study. BMC Pregnancy Childbirth. 2013;13:116.

8. Hailu D, Gulte T. Determinants of Short Interbirth Interval among Reproductive Age Mothers in Arba Minch District, Ethiopia. Int J Reprod Med. 2016;2016:1–17.

9. Sirivong A, Silphong B, Simphaly N, Phayasane T, Bonouvong V, Schelp FP. Advantages of trained TBA and the perception of females and their experiences with reproductive health in two districts of the Luangprabang Province, Lao PDR. Southeast Asian J Trop Med Public Health. 2003;34:919–28.

10. Ismail T, Hamzah T, Hassan M, Mahmood N. Prevalence and factors associated with short birth spacing among Malay women in Kota Bharu, Kelantan, Malaysia. Int Med J. 2008;15:131–6.

11. Atkin LC, Alatorre-Rico J. Pregnant again? Psychosocial predictors of short-interval repeat pregnancy among adolescent mothers in Mexico City. J Adolesc Health. 1992;13:700–6.

12. Dim CC, Ugwu EO, Iloghalu EI. Duration and determinants of inter-birth interval among women in Enugu, south-eastern Nigeria. J Obstet Gynaecol (Lahore). 2013;33:175–9.

13. Fayehun OA, Omololu OO, Isiugo-Abanihe UC. Sex of preceding child and birth spacing among Nigerian ethnic groups. Afr J Reprod Health. 2011;15:79–89.

14. Exavery A, Mrema S, Shamte A, Bietsch K, Mosha D, Mbaruku G, et al. Levels and correlates of non-adherence to WHO recommended inter-birth intervals in Rufiji, Tanzania. BMC Pregnancy Childbirth. 2012;12:152.

15. Muganyizi PS, Mageta D. Does the use of modern family planning promote healthy timing and spacing of pregnancy in Dar es Salaam? Reprod Health. 2013;10:65.

16. Achadi EL, Costello C, Park CB. The contribution of family planning and breastfeeding to birth interval lengths. West Java, Indonesia; 1991.

17. Blackwell AD, Tamayo MA, Beheim B, Trumble BC, Stieglitz J, Hooper PL, et al. Helminth infection, fecundity, and age of first pregnancy in women. Science (80- ). 2015;350:970–2.

18. Dommaraju P. Marriage age and fertility dynamics in India. Calverton, Maryland, USA: Macro International; 2008.

19. Erfani A, McQuillan K. The Changing Timing of Births in Iran: An Explanation of the Rise and Fall in Fertility After the 1979 Islamic Revolution. Biodemography Soc Biol. 2014;60:67–86.

20. Lehrer E. The impact of child mortality on spacing by parity: a Cox-regression analysis. Demography. 1984;21:323–37.

21. Ly CT, Diallo A, Simondon F, Simondon KB. Early short-term infant food supplementation, maternal weight loss and duration of breast-feeding: a randomised controlled trial in rural Senegal. Eur J Clin Nutr. 2006;60:265–71.

22. Nair SN. Determinants of birth intervals in Kerala: an application of Cox’s hazard model. Genus. 1996;52:47–65.

23. Singh R, Tripathi V, Singh K, Ahuja RK, Vani MK, Dwivedi SN. Breastfeeding as a time-varying-time-dependent factor for birth spacing: multivariate models with validations and predictions. World Health Popul. 2012;13:28–51.

24. Upadhyay UD, Hindin MJ. Do higher status and more autonomous women have longer birth intervals? Soc Sci Med. 2005;60:2641–55.

25. Gyimah SO. The dynamics of spacing and timing of births in Ghana. London ON: Population Studies Centre; 2002.

26. Hossain MB, Phillips JF, LeGrand TK. The impact of childhood mortality on fertility in six rural thanas of Bangladesh. New York, NY: Population Council; 2005.

27. van Eijk AM, De Cock KM, Ayisi JG, Rosen DH, Otieno JA, Nahlen BL, et al. Pregnancy interval and delivery outcome among HIV-seropositive and HIV-seronegative women in Kisumu, Kenya. Trop Med Int Health. 2004;9:15–24.

28. Fallahzadeh H, Farajpour Z, Emam Z. Duration and determinants of birth interval in Yazd, Iran: a population study. Iran J Reprod Med. 2013;11:379–84.

29. Youssef RM. Duration and determinants of interbirth interval: community-based survey of women in southern Jordan. East Mediterr Health J. 2005;11:559–72.

30. Hoa HT, Toan N V, Johansson A, Hoa VT, Höjer B, Persson LA. Child spacing and two child policy in practice in rural Vietnam: cross sectional survey. BMJ. 1996;313:1113–6.

31. Adewuyi AA, Isiugo-Abanihe UC. Regional patterns and correlates of birth interval length in Nigeria. Canberra, Australia: International Population Dynamics Program; 1990.

32. Mattison SM, Wander K, Hinde K. Breastfeeding over two years is associated with longer birth intervals, but not measures of growth or health, among children in Kilimanjaro, TZ. Am J Hum Biol. 2015;27:807–15.

33. Adeokun LA. The next child: spacing strategy in Yorubaland (with translations from taped interviews). Philadelphia, PA; 1981.

34. Adeokun LA. Marital sexual relationships and birth spacing among two Yoruba sub-groups. Africa. 1982;52:1–14.

35. de Vera NZ. Birth Spacing Perceptions of Rural Filipinos. J Transcult Nurs. 2007;18:238–46.

36. Dean NR. A community study of child spacing, fertility and contraception in West Pokot District, Kenya. Soc Sci Med. 1994;38:1575–84.

37. Lovel H, Mkandla M, Morley D. Birth spacing in Zimbabwe a generation ago. Lancet. 1983;2:161–2.

38. Millard A V, Graham MA. Breastfeeding and demography in two Mexican villages. East Lansing, Michigan; 1984.

39. van de Walle F, Traore B. Attitudes of women and men towards contraception in Bobo-Dioulasso. Philadelphia, PA; 1986.

40. Ministry of Health Health Education Division. Ministry of Health. Tanzania. Attitudes and beliefs regarding child spacing: focus group discussions with men and women from 6 regions of Tanzania. Dodoma, Tanzania; 1991.

41. Ministere de la Sante. Chad. Focus group discussion report. Attitudes and beliefs about child spacing. “Too many children and no means, it’s desolation].” N’Djamena, Chad; 1992.

42. Indian Market Research Bureau. Institute of Rural and Social Research. Indian focus groups on birth spacing: qualitative study in India. CATALYST Consortium; 2003.

43. Dehne KL. Knowledge of, attitudes towards, and practices relating to child-spacing methods in northern Burkina Faso. J Health Popul Nutr. 2003;21:55–66.
