# Supplementary material for: Factors associated with short birth interval in low- and middle-income countries: a systematic review
Source: BMC Pregnancy Childbirth. 2020 Mar 12;20:156. doi: 10.1186/s12884-020-2852-z (PMC7069040; doi:10.1186/s12884-020-2852-z)
Supplement: Supplementary file 4 — Additional file 4. Studies reporting HR. Table showing the findings of studies reporting HR. [file 12884_2020_2852_MOESM4_ESM.docx]

**Additional file 4. Studies reporting HR**

| Publications | ↑ short birth interval with | Mixed results |
| --- | --- | --- |
| Adewuyi 1990 | previous infant death | Education |
| Blackwell 2015 | NA | Helminth infection |
| Dommaraju 2008 | NA | Age at marriage |
| Erfani 2014 | ↓ education of the mother | NA |
| Fallahzadeh 2013 | ↑ age of marriage and ↑ education of the mother | NA |
| Gyimah 2005 | ↑ age and ↓ education of the mother, rural residence, first birth at earlier age, and previous child loss | NA |
| Hoa 1996 | ↓ education of the mother, farmers as profession, Catholic religion | NA |
| Hossain 2007 | previous childhood mortality |  |
| Lehrer 1984 | NA | Child death |
| Ly 2006 | lack of early short-term infant supplementation | NA |
| Mattison 2015 | ↓ breastfeeding | NA |
| Nair 1996 | ↓ age of the mother, ↑ education of husband and wife, ↓ parity, ↓ breastfeeding, postpartum amenorrhea <6 months | NA |
| Singh 2012 | ↓ breastfeeding | experience of fetal loss, education of women, employment status of women, education of husband, media exposure, survival status of index child, place of residence |
| Upadhyay 2005 | ↓ decision-making autonomy | NA |
| van Eijk 2004 | ↓ age and more education of the mother, being married, negative previous pregnancy outcome (a stillbirth, abortion, or death of a liveborn child), and no-HIV seropositivity | NA |
| Youssef 2005 | ↓ per capita income, ↓ duration of marriage, ↓ survival children, presence of girls only, ↓ breastfeeding, no miscarriage or stillbirths, no use of modern contraception, ↓ age of the mother | NA |
