# Supplementary material for: Factors associated with short birth interval in low- and middle-income countries: a systematic review
Source: BMC Pregnancy Childbirth. 2020 Mar 12;20:156. doi: 10.1186/s12884-020-2852-z (PMC7069040; doi:10.1186/s12884-020-2852-z)
Supplement: Supplementary file 5 — Additional file 5. Quality assessment of the studies. Table showing the quality appraisal scores (two reviewers and average). [file 12884_2020_2852_MOESM5_ESM.docx]

**Additional file 5. Quality assessment of the studies**

| **Author / year of publication** | **Reviewer one** | **Reviewer two** | **Average^a^** |
| --- | --- | --- | --- |
| Quantitative studies reporting HR | | | |
| Hoa 1996 | 5 | 5 | 5 |
| Erfani 2014 | 5 | 4 | 4.5 |
| Blackwell 2015 | 4 | 4 | 4 |
| Dommaraju 2008 | 4 | 4 | 4 |
| Mattison 2015 | 3 | 4 | 3.5 |
| Singh 2012 | 3 | 4 | 3.5 |
| Upadhyay 2005 | 4 | 3 | 3.5 |
| van Eijk 2004 | 3 | 4 | 3.5 |
| Youssef 2005 | 4 | 3 | 3.5 |
| Fallahzadeh 2013 | 4 | 2 | 3 |
| Ly 2006 | 2 | 4 | 3 |
| Gyimah 2005 | 3 | 2 | 2.5 |
| Lehrer 1984 | 2 | 3 | 2.5 |
| Adewuyi 1990 | 2 | 2 | 2 |
| Hossain 2007 | 1 | 2 | 1.5 |
| Nair 1996 | 1 | 2 | 1.5 |
| Mixed methods | | | |
| Dehne 2003 | 4 | 5 | 4.5 |
| Quantitative studies reporting OR/RR | | | |
| Abdullah 2018 | 5 | 5 | 5 |
| Begna 2013 | 5 | **5** | 5 |
| Hailu 2016 | 5 | 5 | 5 |
| Chirwa 2014 | 4 | 5 | 4.5 |
| de Jonge 2014 | 4 | 5 | 4.5 |
| Muganyizi 2013 | 4 | 5 | 4.5 |
| Achadi 1991 | 3 | 5 | 4 |
| Exavery 2012 | 4 | 4 | 4 |
| Ismail 2008 | 4 | 4 | 4 |
| Todd 2008 | 4 | 4 | 4 |
| Atkin 1992 | 4 | 3 | 3.5 |
| Dim 2013 | 4 | 3 | 3.5 |
| Fayehun 2011 | 3 | 4 | 3.5 |
| Franca-Junior 1985 | 3 | 3 | 3 |
| Ngianga-Bakwin 2005 | 3 | 3 | 3 |
| Sirivong 2003 | 3 | 3 | 3 |
| Qualitative studies | | | |
| De Vera 2007 | 5 | 4 | 4.5 |
| Chad Ministry of Public Health 1992 | 2 | 5 | 3.5 |
| Dean 1994 | 4 | 3 | 3.5 |
| Kiluvia 1991 | 2 | 5 | 3.5 |
| Social & Rural Research Institute 2003 | 2 | 5 | 3.5 |
| Adeokun 1982 | 2 | 2 | 2 |
| Lovel 1983 | 2 | 2 | 2 |
| Van de Walle 1986 | 2 | 2 | 2 |
| Adeokun 1981 | 2 | 1 | 1.5 |
| Millard 1984 | 2 | 1 | 1.5 |

**^a^** 0-1= low quality; 2-3= medium quality; 4-5= high quality
